# Deciphering mycobiota and its functional dynamics in root hairs of *Rhododendron campanulatum* D. Don through Next-gen sequencing

**DOI:** 10.1038/s41598-024-61120-4

**Published:** 2024-05-04

**Authors:** Nafeesa Farooq Khan, Sheikh Sajad Ahmed, Mukhtar Iderawumi Abdulraheem, Zafar Ahmad Reshi, Abdul Wahab, Gholamreza Abdi

**Affiliations:** 1 Biological Invasion Lab, Department of Botany, University of Kashmir, Srinagar, Jammu & Kashmir 190006 India; 2 Department of Electrical Engineering, Henan Agricultural University, Zhengzhou, 450002 China; 3 Henan International Joint Laboratory of Laser Technology in Agriculture Science, Zhengzhou, 450002 China; 4 University of Chinese Academy of Sciences, Beijing, 100049 China; 5 Shanghai Center for Plant Stress Biology, CAS Centre for Excellence in Molecular Plant Sciences, Chinese Academy of Sciences, Shanghai, 200032 China; 6 Department of Biotechnology, Persian Gul Research Institute, Persian Gulf University, Bushehr, 75169 Iran

**Keywords:** Microbial, Metabarcoding, *Rhododendron campanulatum*, NextGen sequencing, Biological techniques, Biotechnology, Developmental biology

## Abstract

The Himalayas provide unique opportunities for the extension of shrubs beyond the upper limit of the tree. However, little is known about the limitation of the biotic factors belowground of shrub growth at these cruising altitudes. To fill this gap, the present study deals with the documentation of root-associated microbiota with their predicted functional profiles and interactions in the host *Rhododendron campanulatum,* a krummholz species. While processing 12 root samples of *R. campanulatum* from the sites using Omics we could identify 134 root-associated fungal species belonging to 104 genera, 74 families, 39 orders, 17 classes, and 5 phyla. The root-associated microbiota members of Ascomycota were unambiguously dominant followed by Basidiomycota. Using FUNGuild, we reported that symbiotroph and pathotroph as abundant trophic modes. Furthermore, FUNGuild revealed the dominant prevalence of the saptroptroph guild followed by plant pathogens and wood saprotrophs. Alpha diversity was significantly different at the sites. The heatmap dendrogram showed the correlation between various soil nutrients and some fungal species. The study paves the way for a more in-depth exploration of unidentified root fungal symbionts, their interactions and their probable functional roles, which may serve as an important factor for the growth and conservation of these high-altitude ericaceous plants.

## Introduction

The treeline represents the upper altitudinal or latitudinal limit beyond which plant species do not grow as trees. In the context of the prevailing climate change scenario, when the vulnerability of treeline species in mountain ecosystems is presumed, a significant number of species are presumed to shift their geographic distributions to match the climatic conditions which have sustained their abundance till now^1–4^. From time to time, habitats such as high latitudes and altitudes have been reported as extremely vulnerable to rising temperatures^5–7^ yet studies in treelines mainly emphasize altered abiotic variables such as temperature, precipitation, and soils^8–11^. In past few years empirical studies^12–16^ have suggested role of biotic interactions as a key to understanding current species distributions, abundances, and range limits. However, the composition pattern of belowg-round organisms remains understudied^17^ even though these belowground organisms may have a significant role in supporting these treeline species in harsh high-elevation conditions^18–20^. In fact, there are numerous microbiota present near the treeline of which a rough estimate of 50–90% of total soil microorganisms biomass comprise of fungi^21^. While fungi in the treeline are mostly inconspicuous, they are known to play an indispensable role in the cyclization of organic matter and in the stream nutrients through various trophic levels in ecosystems^22–25^ in exchange for their own carbon needs. Although microbial assemblages of many dominant treeline-forming species have been documented earlier^26,27^ relatively few studies are available on the root associated mycobiota of the *Rhododendron campanulatum* which is a widespread woody and krummholz plant species in the Kashmir Himalayan region belonging to Ericaceae, and often the identification of its root mycobiome is restricted to culture dependent studies^28–30^. This plant species grows in low nutrient sites at high latitude or high elevations with a unique mycorrhizal association, called ericoid mycorrhiza (ERM) which is essential for its growth and survival in nutrient-poor and stressful environments^30–32^. While some studies have identified the arbuscular mycorrhizal status of the Rhododendron species^33^. Little is known about the composition of the microbial diversity of this plant species. To improve our understanding of the below-ground biota, we analysed the below-ground compositional changes in sites spread across two mountain systems in Kashmir Himalaya. We also aimed to pair our results with soil nutrient content (N, P, K, OC, EC, pH, Ca, Mg) to investigate the relationships between below-ground microbial taxa and soil nutrients. Using DNA metabarcoding and targeting the ITS2 regions of ribosomal DNA, we obtained quantitative information about the below-ground mycobiota.

The study evaluated the diversity, guild structure, and trophic modes of root associated fungi in *R. campanulatum* in Kashmir Himalaya, employing morpho-molecular approach of characterization to understand how and what kind of species interactions affect species diversity at treelines. Consequently, we hypothesised that the microbial composition across sites does not change. This theoretical basis of microbial composition across sites does not change stems from:

1. Similar environmental conditions such as pH, humidity, temperature, and nutrient availability.
2. The plant shows limited dispersal because it grows around the same altitude in two study sites.
3. Additionally, the functional role of microbes present in this habitat can reach functional redundancy and successional stability.

To order to test this hypothesis, we precisely asked the following research questions. Whether (i) there is a compositional change in the soil biota, in terms of both taxonomic and functional groups, between the study sites; (ii) the changes in taxonomic and functional groups of roots associated fungi are correlated with changes in soil nutrients, and (iii) the fungal diversity changes with altitudinal variations.

## Material and methods

### Description of the sampling site and sample collection

This study was carried out in the Kashmir Himalayan region in geographical coordinates of 75°51′02″ E and 33°58′11″ at Sinthan top site and 74°32′36″ E and 34°03′55″ N at Apharwat. The two sites were comparable in terms of altitude and exposure. Field sampling included collection of 250 g rhizospheric triplicate soil samples and hairy tertiary root samples from natural and actively growing plants of *R. campanulatum* (diameter < 2 mm)^34^ of roughly the same developmental stage along elevational gradients (150-200 m) in two alpine locations. Although the sites were snow covered from December to early July, sample collection was carried out between July and August. Along the elevational gradient, from thick vegetation to uppermost alpine zone, sampling was carried out in triplicates of plants < 40 cm in height and 30 m apart at four elevations (3396 and 3552 amsl at Sinthan top and 3610 and 3800 amsl at Apharwat). A total of 12 root samples were processed for molecular characterization of the microbiome. Samples were taken from a depth of 30 cm using^35^ soil core method and 3 soil cores from one individual were pooled to make one composite sample. The collected roots were aseptically transferred into separates sterile zip-lock plastic bags labelled and transported to the laboratory using cool packs. A portion of the roots was processed for morphological identification using taxonomic keys under the Olympus CX51, Japan microscope using^36^ key 1987–1995 (DEEMY). The clear and sorted root samples were kept in 50 ml centrifuge tubes with CTAB buffer at − 20 °C until processed for molecular analysis. For identification of plant species, the voucher sample specimen was deposited to Kashmir University herbarium under accession KASH-3714.

### Nutrient analysis

Dried and crushed 250gm soil samples (dried at 100 °C for 48 h) were passed through 2.0 mm sieve to remove roots and other aggregate matter. The soil chemical analysis was performed at the division of soil science, SKUAST-K Shalimar. pH was determined electrochemically with the help of glass electrode pH meter, as suggested by Jackson 1973. Exchangeable cations were extracted using 1 mol L^−1^ neutral ammonium acetate solution and quantified using the Kjeldahl method to measure the cation exchange capacity (CEC)^37^. The organic carbon of the soil samples was determined using the Walkley and Black method as outlined by^38^. Total nitrogen of the soils was determined using the Kjeldahl’s method following H_2_SO_4_ acid digestion method as suggested by^38^. The available K was determined from NH_4_OAc. (pH, 7.0) extract as described by^38^. The extract was analysed for available K by a flame analyzer at 589 nm. The available phosphorus was extracted from the soil with 0.5 M NaHCO_3_ (Olsen’s Method) at pH 8.5 and molybdo-phosphoric blue colour method of analysis was used for determination. Calcium and magnesium were estimated using atomic absorption spectrophotometer (AAS 4141) protocol using^38^. All quantified soil properties are given in Supplementary Table S1.

### Root length colonization

Hairy root samples were cut into pieces and soaked in 40% KOH for 10 min after rinsing with 1% HCl, staining and distaining using^39^ protocol. 1200 pieces of roots were screened for the extent of mycorrhizal infection at magnification of 10 × and 40x. For calculation of the fungal colonization rates, the total number of colonized root segments and the total number of root segments studied were recorded. The percentage was calculated using the formula:-

$$RLC \left(\%\right)=\frac{Total number of root segments colonized}{Total number of root segments studied}\times 100$$

### DNA extraction, PCR amplification

12 root samples were homogenized in liquid nitrogen using the CTAB method to obtain genomic DNA using a standard protocol^40^. The product obtained was checked on a 1.2% agarose gel and its concentration was checked on Qubit 4 fluorimeter (Invitrogen). Dilution in genomic DNA was made wherever required to obtain a minimum DNA requirement of 50 μl per PCR reaction. Amplification was carried out for each sample using the fungal specific marker pair ITS1 (TCCGTAGGTGAACCTGCGG) and ITS4 (TCCTCCGCTTATTGATATGC) using and Fusion high fidelity Taq in a 20 μl reaction volume. The PCR reaction consisted of Genomic DNA 2 μl, 10X PCR buffer 2 μl, 2 mM dNTPs (Sigma) 0.5 μl, Forward Primer Sigma 0.4 μl, Reverse Primer 0.4 μl, DMSO 0.5 μl, BSA 0.5 μl, MiliQ water 13.5 μl and Taq polymerase 0.2 μl. The set standardised conditions used for PCR were 95^◦^C for 4 min (Denaturation), 30 cycles of 30 s at 95^◦^C (denaturation), 60^◦^C for 30 s (annealing) and 72^◦^C for 1.30 s (extension), followed by 5 min at 72^◦^C followed by 4 degree for infinity. The amplified products were checked on a 0.7% agarose gel and viewed in the Gel Doc System to confirm amplicon amplification.

### Nanopore library preparation

Each sample was concentrated by magnetic beads using magnetic separator for nanopore library preparation using the version of the amplicon library preparation sequencing kit, which was then purified by 1 × Agencourt AMPure XP SPRI magnetic beads and barcoded by the NEBNext End repair/dA-tailing component then ligation to protein-linked adapters AMX (Oxford Nanopore Technologies, Oxford, UK) using the NEBNext Quick Ligation component kit. All the PCR products barcoded by barcoding kit were then pooled according to the SQK-LSK108 protocol for the 1D PCR barcoded amplicon and stored on ice until loaded into a flow cell for sequencing. Sequencing was carried out on MinION flow cells (FLO-MIN106D R9.4.1) (Oxford Nanopore Technologies, Oxford, England) sequencer at the Yaaz Genomics laboratory, Coimbatore, India.

### Bioinformatics and data analysis

MinKNOW version 2.0 was used for live basecalling. The barcodes and adapters were removed by using Porechop (https.//github.com/rrwick/Porechop). The FASTQ files were analysed on the Nanopore EPI2ME platform with a minimum qscore of > 10. Reads were assigned fungal identification in a real-time analysis with cloud-based fungal identification facilitated via What is In My Pot?’ (WIMP) workflows application from the EPI2ME platform (Oxford Nanopore Technologies Ltd, UK), allowing the identification of the fungal species a few minutes after the run started. The EPI2ME platform quality filtered and clustered sequences into operational taxonomic units (OTUs) at 97% similarity^41^. The sequence data generated for this study is available from NCBI SRA under the accession number PRJNA669306.

OTU tables summarizing the abundance of fungal taxa in each sample were calculated separately. The fungal OTUs were then taxonomically analysed for the identification of ecological guilds or “functional groups” using FUNGuild (https://github.com/UMNFuN/FUNGuild)^40^. The observed and predicted (Chao1) numbers of OTUs per sample, as well as the equitability of their abundances (Simpson’s and Shannon’s diversity indices) were calculated using Microbiome analyst^42^. The read data was further analysed in Kaiju (https://bioinformatics-centre.github.io/kaiju/) online tool for easy taxonomic classification and assessment of the diversity of the two study regions^43^. Interactive venn (https://www.interactivenn.net/), an online tool, was used to show common and specific taxa among the sites and along elevational gradient^44^. Two of the samples had poor quality reads, and as such were filtered out from downstream analysis.

## Results

### Morphotyping and Root length colonization

**Figure 1:**
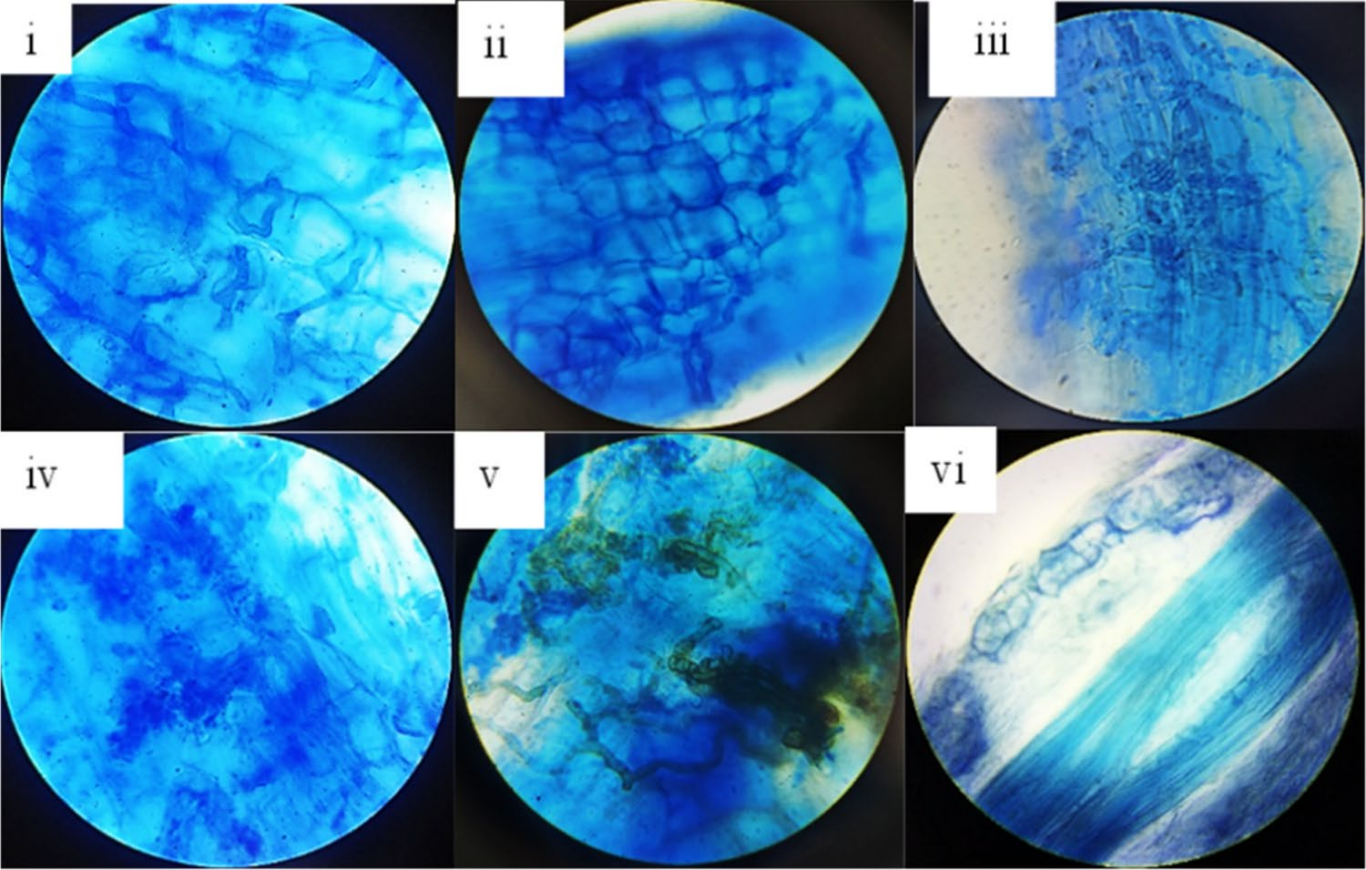
ECM morphotypes from roots of *R. campanulatum.*

We reported a rare occurrence of ECM morphotypes in *R. campanulatum* Fig. 1*.*

A small percentage of morphotypes were observed in *R. campanulatum* using the Agerer’s taxonomic key which constituted a total of 20 morphotypes. The observed morphotypes (Supplementary Figure S1) and their description are provided in Supplementary Table S2. Moreover, through root length colonization we showed the presence of characteristic intracellular and intercellular ericoid hyphal coils inside the root cortical cells along with the incidence of dark septate hyphae within these cells, in addition to ectomycorrhizae (morphotypes). No typical ectomycorrhizal structures were formed in epidermal cells of the root tips (Fig. 1). Hyphae were not observed within the root stele.

### Richness and diversity patterns of root-associated fungi

**Figure 2:**
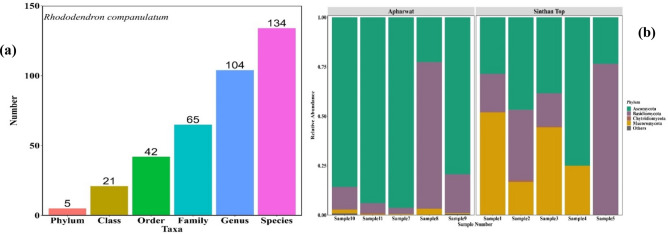
(**a**) Taxonomic classification of identified fungi. (**b**) Relative diversity of phylum identified.

Based on MinION amplicon sequencing, using 97% similarity cut-off, the fungi identified in the present study were clustered into 134 species, 104 genera, 74 families, 39 orders, 17 classes, and 5 phyla (Fig. 2a) (to normalize read depth) with 502 average counts of reads per sample. The total number of reads obtained from Oxford Nanopore MinION sequencing amounted to 26,465 reads. The samples collected resulted in 11,712 reads and 14,753 reads at site 1 and site 2 respectively (Supplementary Table S3) and were assigned various taxa. Of all the reported phyla, the diversity was primarily dominated by the phylum Ascomycota (Fig. 2b).

**Figure 3:**
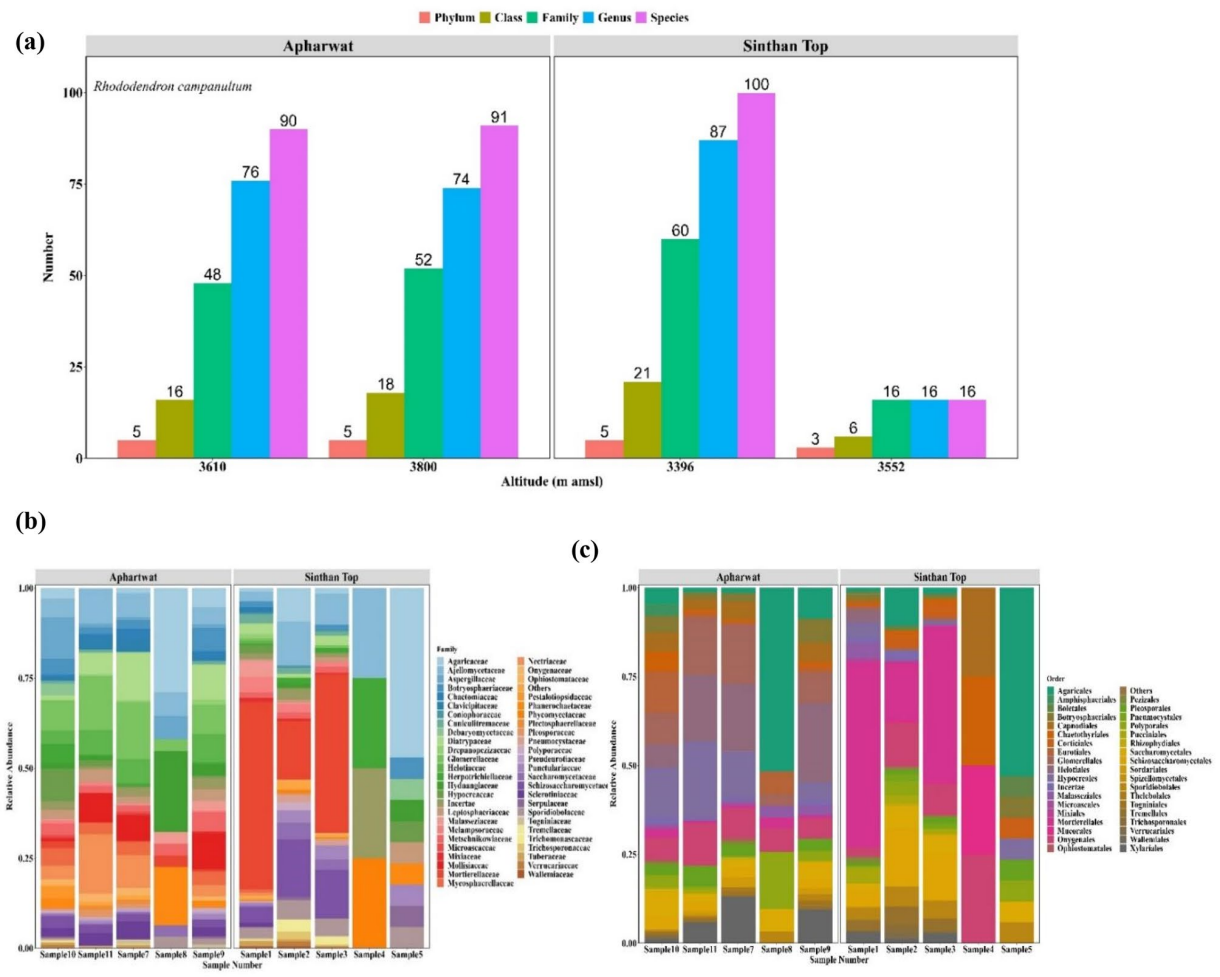
(**a**) Number of root-associated fungal taxa along the altitudinal gradient at Apharwat and Sinthan-top. (**b**) Comparative diversity analysis of taxa found attwo study sites. (**c**) Depicts the order level diversity from samples from two study site Further, genus *Tremella, Coniophora, Wallemia, Vanderwaltozyma, Fomitiporia, Heterobasidion, Dactylellina, Capronia, Cordyceps, Zygosaccharomyces, Schizophyllum, Naumovozyma, Ascoidea* and *Saccharomyces* were specific to Sinthan top (Fig. 4a) while the genera *Wickerhamomyces, Auricularia, Coniosporium, Histoplasma, Kazachstania, Lodderomyces, Microsporum, Nannizzia, Neurospora, Pyrenophora, Sclerotinia* and *Thielavia* were specific to Apharwat (Fig. 4a) In addition, our findings based on the Chao1 richness estimator, ACE richness estimator, Simpson dominance, and Shannon diversity index from each sample showed that alpha diversity patterns significantly varied between sites Fig. 4b. Additionally, the highest value of Shannon was in sample 2 with value of 3.15 while lowest value was obtained from sample 4 i.e. 1.386 at Sinthan top. Likewise, the highest value of Shannon was in sample 9 value 3.79 while the lowest value was in sample 7 values 2.12 at Apharwat. For comparisons, Shannon, Simpson, Fisher alpha values and other indices per sample data is presented in Supplementary Table S4. Furthermore, PCoA based on Bray–Curtis dissimilarities and PERMANOVA analysis (p < 0.05) (Fig. 4c) showed a clear difference between Apharwat and Sinthan top samples confirming the different composition of the mycobiota between two sites.

The composition of fungi evaluated at the phylum rank as presented in Fig. 2b shows the overall predominance of Ascomycota constitute 53.9% of total diversity while Basidiomycota constituted 27.4%. Comparatively, the highest number of species were obtained at Apharwat (species 108, genus 90, family 60, order 35, class 15 and phylum 5) than at Sinthan top (species 105, genus 92, family 63, order 38, and class 17 and phylum 5) (Fig. 3a).

Evidently, data showed that with increase in elevation the diversity of fungal taxa showed a sharp decline at Sinthan top while the fungal assemblage remained more or less similar for two altitudes of Apharwat, displaying no pattern in diversity (Fig. 3a). The perusal of data indicates those 5 phyla, 15 classes, 34 orders, 49 families, 78 genera, and 79 species were shared by two study sites. There were 2 classes, 4 orders, 14 families, 14 genera and 26 species specific to the Sinthan top and 1 order, 11 family, 12 genera, and 29 species specific to the Apharwat, respectively (Supplementary Figure S2). At the order rank of the hierarchy, Agaricales and Mortierellales were the most dominant orders. Among the reported fungal families, Ascoideaceae, Bondarzewiaceae, Coniophoraceae, Cordycipitaceae, Dermateaceae, Fomitopsidaceae, Hymenochaetaceae, Phaeosphaeriaceae, Schizophyllaceae, Sporocadaceae, Teratosphaeriaceae, Tremellaceae, Trichocomaceae and Trichosporonaceae were specific to Sinthan while Auriculariaceae, Coniosporiaceae, Drepanopezizaceae, Fibroporiaceae, Hydnangiaceae, Mollisiaceae, Pestalotiopsidaceae, Pneumocystaceae, Wickerhamomycetaceae, Sordariaceae and Trichosporonaceae were specific to Apharwat (Fig. 3b). At the order level of classification, Wallemiales Hymenochaetales, Russulales and Orbiliales were specific to Sinthan top, whereas a single order Auriculariales was specific to Apharwat (Fig. 3c).

**Figure 4:**
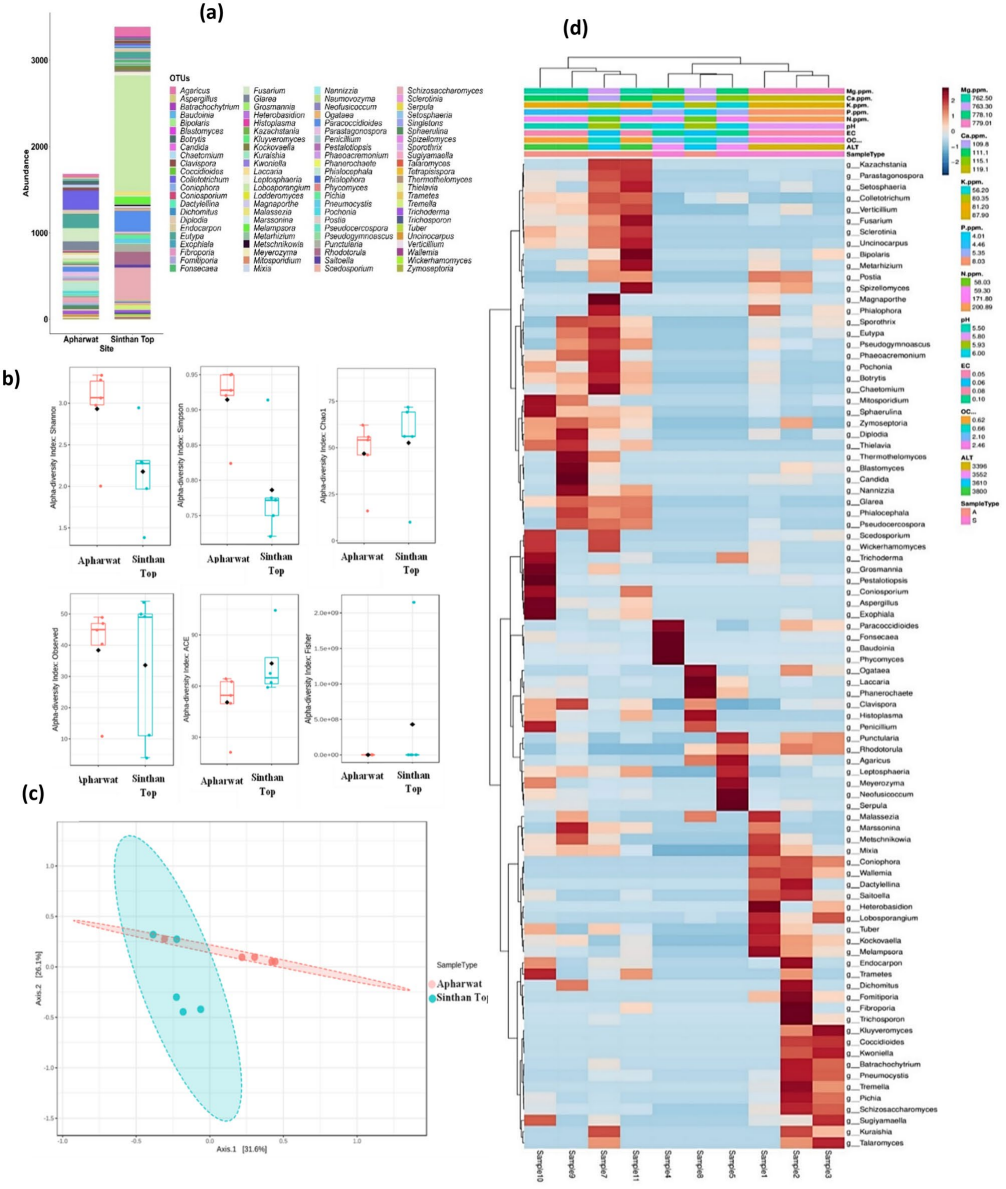
(**a**) Relative abundance of dominant genera at study sites. (**b**) Estimates of diversity indices at two study sites. (**c**) PCoA based on Bray–Curtis dissimilarities and PERMANOVA analysis (p < 0.05). (**d**) Distance correlation differences at the order level community composition were correlated with differences in environmental factors.

### Dendrogram and nutrient analysis

In this study, distance correlation differences at order level community composition correlated with differences in environmental factors showed some taxa correlated with environmental variables. In depth analysis revealed that high K, P, N, EC, OC and low pH had the strongest correlation with orders Orbiliales, Hymenochaetales, Tremallales, Schizomycetales, Pneumocystales, Rhizophydiales, Trichosporonales, Boletales, Wallemiales, Rusulales, Mixales, Verrucariales, Pezizales while low K and high P, N, pH, EC, OC showed correlation with orders Onygenales, Chaetothyriales, Capnodiales, Mucorales, Boletales, Hypocreales, Agaricales, Sporidiobolales, Corticales and Pleosporales. Moderate levels of K, P, N and high pH, EC, OC affected diversity of Eurotiales, Amphisphaeriales, Coniosporales, Helotiales, Microscacales, Sacchromycetales, Hypocreales, Glomeralles, Ophiostomiales, Spizellomycetales and Botryosphaeriales. Further the diversity of orders, Magnoporthales, Theleborales, Xylariales, Sordariales, Togniniales was significantly correlated with high K, pH, EC and low P, N, OC. while low K, P, N, OC and high pH and EC. Overall, there was a significant difference in the structure of the fungal community with respect to organic carbon, pH, EC, nitrogen, phosphorus and potassium, which varied significantly between sites (T.test P < 0.05) (Fig. 4d).

### FUNGuild classification of guilds and trophic modes

Of the 134 identified species, 91 species were assigned different trophic modes and guilds according to FUNGuild parsing. Most identified species belonged to animal pathogens, arbuscular mycorrhizal fungi, ectomycorrhizal fungi, lichenized fungi, mycoparasites, plant pathogens, undefined root endophytes, undefined saprotrophs, unassigned fungi, and wood saprotrophs with a difference confidence level (Supplementary Table S5).

**Figure 5:**
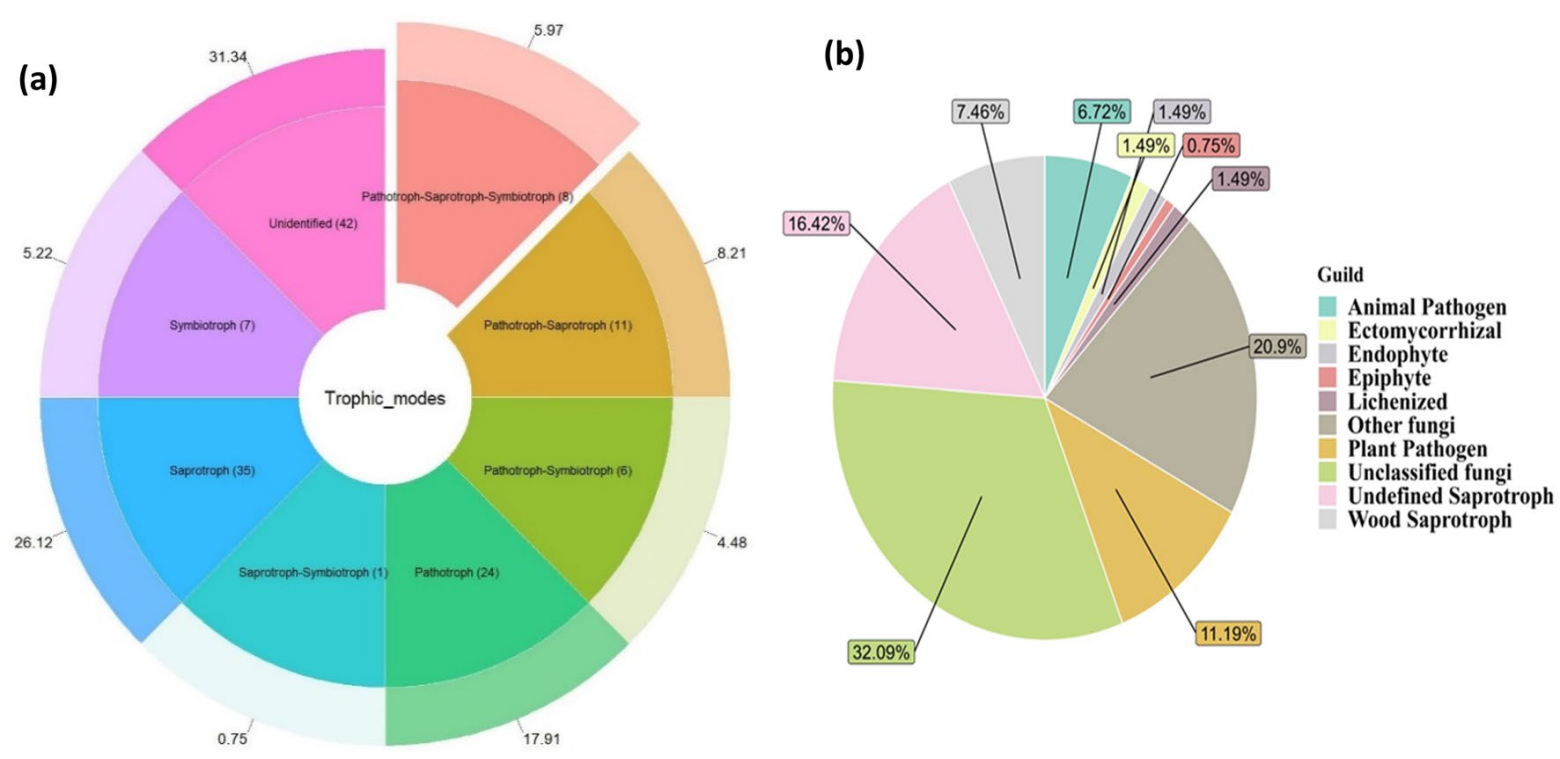
(**a**) Classified trophic modes using FUNGuild. (**b**) FUNGuild classified trophic modes.

The most dominant fungal functional type in the data set was Saprotrophs with 35 taxa, followed by pathotroph (24 assigned taxa). Only 11 assigned taxa belonged to the pathotroph-saprotroph trophic mode. In comparison to other trophic modes Pathotroph-saprotrophs symbiotroph comprised 7 taxa. Similarly, 7 taxa belonged to symbiotroph trophic mode (Fig. 5a).A total of 43 taxa remained unknown for their functional attributes, and the remaining taxa were assigned more than one possible trophic mode (Fig. 5b).

### The core microbiota of the host

**Figure 6:**
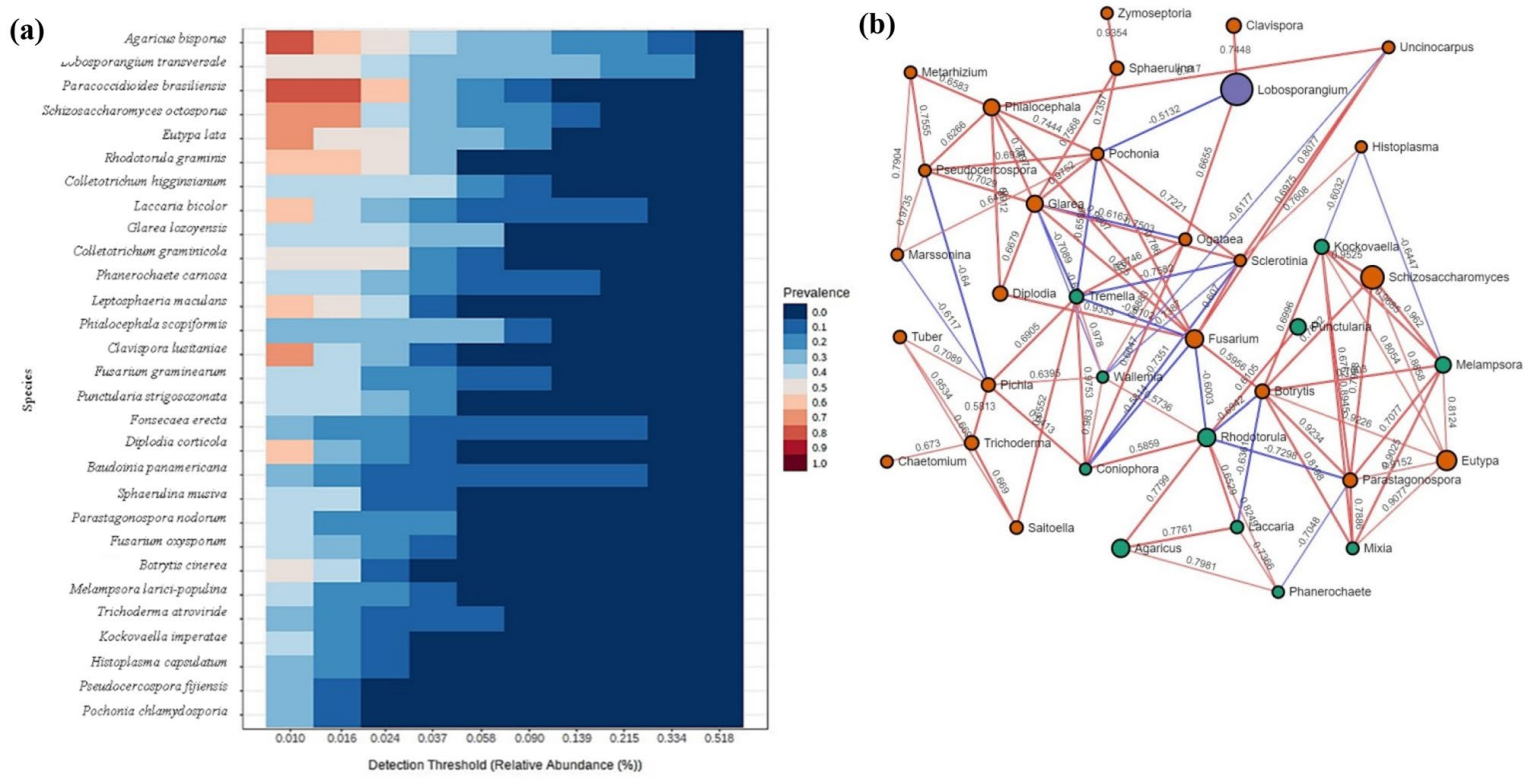
(**a**) Core microbiome associated with roots of *R. camapnulatum.* (**b**) SparCC analysis showing co-occurring networks of the abundant taxa shared by all the samples at a correlation threshold of 0.3. The size of the circle corresponds to average relative abundance and colour corresponds to the fungal phyla.

The core microbiota of this plant constituted species belonging to the genus *Agaricus, Lobosporangium, Paracoccidioides, Schizosaccharomyces, Eutypa, Rhodotorula, Colletotrichum, Laccaria, Phialocephala, Clavispora, Fusarium, Punctularia, Fonsecaea, Diplodia, Baudoinia, Sphaerulina, Parastagonospora, Botrytis, Melampsora, Trichoderma, Kockvaella, Hostoplasma, Pseudocercospora, Pochonia* with a prevalence threshold approximation of 75% and relative abundance of 0.010% in all samples (Fig. 6a).

### Sparse correlations for compositional data

The co-correlation analysis showed co-occurring networks of the abundant taxa shared by all the samples at a correlation threshold of 0.3 using the Sparse Correlations for Compositional data (SparCC). A total of 36 nodes (co-abundance groups-CAGs) were formed from all group comparisons showing all possible positive and negative but significant interactions (Fig. 6b).

The majority of nodes belonged to ascomycota followed by basidiomycota and Mucuromycota and the microbial dysbiosis index was 0.6878. Among abundant fungal taxa, *Zymoseptoria, Sphaerulina, Clavispora, Uncinocarpus, Lobosporangium, Phialocephala, Pochonia, Pseudocercospora, Glarea, Ogataea, Sclerotinia, Kockvaella, Schizosaccharomyces, Melampsora, Punctularia, Laccaria, Fusarium, Botrytis, Parastagonospora, Mixia, Eutypa, Rhodotorula, Phanerochaete, Agaricus, Tremella, Coniophora, Wallemia, Trichoderma, Saitoella, Pichia, Chaetomium, Tuber, Diplodia, Marssonina, Metarhizium, Hostoplasma* showed significant positive and negative interactions in these taxa with p-values 0.05 using a bootstrap procedure with 100 random permutations.

## Discussion

The present study revealed a total of 20 ECM root morphotypes with uncommon instances. A limitation to morphological data is the lack of comparison of reported ECM morphotypes in this plant species due to the presence of fine root hair. Unlike the present study, previous studies have shown preferential screening of root hair that only shows ERM colonization, whereas higher order roots are seen to form root morphotypes. In addition to distinguishable intracellular ERM hyphal coils inside rhizodermal cells in root hair, other fungi, for example, DSE (Dark Septate Endophyte), ECM fungi, AM fungi, saprophytic or pathogenic fungi, constituted the root fungal community in plant species and were considered to play either positive or negative roles for their hosts in the natural environment. These fungal taxa were similar to observations in other ericaceous plants^30,45–47^, and their functions on roots of ericaceous plants should be evaluated in further studies. In contrast to the present study,^48^ reported absence of ECM and AM in the roots of Ericaceae. The degree of internal colonization by arbuscular mycorrhizal fungi could depend on differences in root morphology. Moreover, colonization rates are known to vary with the altitude, humidity, precipitation, temperature, and plant community^49–52^. We also reported the presence of DSE in the tertiary roots of the host and its presence has previously been reported from antarctic and sub-arctic sites that form intracellular mycelium and microsclerotium primordium^53,54^. As in the present work, these DSE structures have been reported from similar environmental conditions, such as alpine and arctic habitats, confirming the mutualistic role of these fungi^55,56^. Though we noticed that many studies have documented the ericoid mycorrhizal status of family Ericaceae^57,58^, the work described here represents the first attempt to identify and infer the taxonomic affinities of the root associated fungi in *R. campanulatum* using both morpho-molecular methods*.* Although both culture-based and culture-independent methods introduce potential bias^59–61^, a combination of both types of approach is preferred to reduce bias in investigations of similar nature^33,62–67^.

Analysis of nuclear ribosomal DNA sequences suggests the roots of *R. campanulatum* harbour diverse assemblages of mycorrhizal and non-mycorrhizal fungal symbionts. Although the diversity obtained is less than those obtained from roots other treeline plant species, including other species of the genus *Rhododendron*. The difference in diversity can also be attributed to the limited number of samples for study sites reflected through rarefaction curves (Supplementary Figure S3), which in addition to showing insufficient samples at one site shows observed OTU richness was higher at Apharwat than at Sinthan top. Furthermore, the alpha diversity differed significantly between the sites. Compared to the Sinthan top, the highest diversity was recorded for Apharwat which may be attributed to the soil parameters that varied significantly between study sites with the highest value of shannon index 3.7 approx. In addition, Ascomycota and Basidiomycota that were dominant phyla in this study, phylum Glomeromycota and Chytridiomycota were found relatively less abundant similar to those observed in *Rhododendron simsii*^68^. Furthermore, the proportion of fungal group richness and evenness decreased with increasing in elevation at the Sinthan top while it remained almost the same for the Apharwat site. This pattern of fungal assemblage is similar to^69^ who observed soil fungal richness and evenness decreased with the increasing elevation gradient, while at a large-scale elevational mountain gradient^70^, found contrasting results showing that microbial richness was not correlated with elevation. However, some fungal taxa were common to the root systems of *R. campanulatum* in two investigated sites with varied relative abundance while others were present only in a single site. The contrasting diversity patterns between two sites is attributed to the soil C/N ratio, moisture, pH, total nitrogen (TN) and temperature^71^. In this study, the results show that soil physico-chemical factors had obvious effects on fungi diversity. Furthermore, the data indicates several unnamed fungal species are present that apparently belong to the group Ascomycota and may also be important associates of ericoid mycorrhizal root associates which need further examination. The Shannon diversity index of the fungi for the plant species ranged from 1.386 to 3.15 at Sinthan top and from 2.127 to 3.795 at Apharwat, respectively. This study has shown that rDNA ITS-based techniques can be used effectively as an initial screen for the composition pattern of root associated fungi. Although the relatively small number of reference sequences currently available makes detection and identification of unknown clones problematic at present, this situation will improve as greater number of ITS fungal sequences becomes available. The order Helotiales, Hypocreales, Sebacinales, and Chaetothyriales, and the family Herpotrichiellaceae, Myxotrichaceae, and Sebacinaceae, which have been found in hair roots of many ericaceous plants^72–74^. Furthermore, some Ascomycetous fungi forming ErM include mostly belong to class Leotiomycetes and some Basidiomycetes in the Serendipitaceae^75^.

FUNGuild analysis showed that the endophytes of the culture-independent approach potentially comprised various functional groups. Based on the FUNGuild inferred lifestyle of obtained fungi, the study indicates greater abundance of saprotrophs in the dataset with few others showing numerous life modes such as pathotroph-saprotrophs, pathotroph-saprotrophs symbiotroph. The prevalence of Saprobes and symbionts in the *Rhododendron* rhizosphere has been reported by^76^. Furthermore, the higher abundance of saprotrophs is related to the presence of free-living saprotrophic fungi that share fundamental decomposition niches with mycorrhizal fungi and compete with root associated mycorrhizae for Nitrogen in limited availability of Nitrogen^24^. We also found that pathogenic fungi were abundant in all sites. This finding is similar to findings of^77^ from arctic. Furthermore, through our findings, we provide a better understanding of the diversity of the root inhabiting pathogen in *R. campanulatum* which can drived the successful movement or range-expansion^78,79^ of species into novel environments. Although we acknowledge that our analyses may not have detected important variation in other aspects of the potential non-fungal pathogens, this study provides novel insights into the rhizosphere microbiome of *R. campanulatum*.

By thorough data analysis, we also explored the core microbiota associated with the host plant. A total of 24 genera formed the core microbiome of the study plant. This highlights that several taxonomic groups of fungi potentially play a key role in synchronizing community processes of temperate and/or subtropical forests. Core symbolises very specific host microbial associations and given the benefits of below-ground plant–fungus symbioses in terrestrial biosphere^80,81^, identifying fungal species that potentially have great impacts on community scale processes by such microbiome interactions will provide crucial insights into the effectiveness, conservation and restoration of treeline ecosystems.

Spar CC analysis revealed complex positive and negative significant interactions among 36 taxa identified. Sparcc is a robust and efficient method for inferring correlation networks from large microbiome datasets based on iterative approximation and log ratio transformed data. A plethora of methods are already known to study microbial interaction even in environmental samples, but sparcc is beneficial when analysing datasets with 500 or fewer OTUs which was previously intractable, yet is likely to become commonplace in modern cohort studies. However, the precise role and activities of fungal symbionts remain unexplored and insights into root associated microbiome interaction are compounded by its complexity^82^. Together identification of core microbiome and taxa cooperative and competitive intra-microbial interactions serves as important factors for conservation of such species.

## Conclusions 

The study of ericaceous root associated symbionts in the treeline ecosystem of the Kashmir Himalaya has revealed a wide diversity of fungi associated with the study plant species. The study is a comparative profile of the rhizospheric fungal community of the endemic plant *R. campanulatum* from four altitudinal zones in two regions of Kashmir Himalaya. While there were few specific taxa at each site, the proportion of shared taxa was quite high. The core microbiome consisted of few indicator taxa that showed prevalence of 75% and no preferential choice of specific taxa. The study gave insight into the myriad functional interactions of fungal. In addition, the study is the first report to document the microbial assemblage of endemic Krummholz shrub species from the region. The plant showed dominance of the phylum Ascomycota and preferred few ectomycorrhizal associates. Few taxa showed preferential occurrence with the various nutrient content while few other taxa formed the core microbiome of the host plant species. We also found certain positive and negative interactions using sparcc between abundant microbiota in the study. In general, the study is a first attempt to document the mycobiota associated with the mycorrhizal and non-mycorrhizal root of *R. campanulatum* from Kashmir Himalaya. The study encourages and permits the carrying out of re-synthesis experiments and the evaluation of nutrient transfer systems to resolve the ability of some mycorrhizal strains occurring in mycorrhizae symbiosis with this plant species to improve the growth and conservation of this ericaceous species.

### Supplementary Information

Supplementary Information.

## Data Availability

The datasets generated and analysed during current study are available in the National Center for Biotechnology Information (NCBI), https://www.ncbi.nlm.nih.gov/sra/?term=PRJNA669306.

## References

[1] Chen IC, Hill JK, Ohlemüller R, Roy DB, Thomas CD (2011). Rapid range shifts of species associated with high levels of climate warming. Science.

[2] Corlett RT, Westcott DA (2013). Will plant movements keep up with climate change?. Trends Ecol. Evol..

[3] Du C (2017). Variations in bacterial and fungal communities through soil depth profiles in a *Betula albosinensis* forest. J. Microbiol..

[4] Bryn A, Potthoff K (2018). Elevational treeline and forest line dynamics in Norwegian mountain areas—a review. Landsc. Ecol..

[5] Serreze MC, Barry RG (2011). Processes and impacts of Arctic amplification: a research synthesis. Global Planet Change.

[6] Pecl GT (2017). Biodiversity redistribution under climate change: Impacts on ecosystems and human well-being. Science.

[7] Pepin NC (2022). Climate changes and their elevational patterns in the mountains of the world. Rev. Geophys..

[8] Bahram M, Põlme S, Kõljalg U, Zarre S, Tedersoo L (2012). Regional and local patterns of ectomycorrhizal fungal diversity and community structure along an altitudinal gradient in the Hyrcanian forests of northern Iran. New Phytol.

[9] Elith J, Leathwick JR (2009). Species distribution models: ecological explanation and prediction across space and time. Annu. Rev. Ecol. Evol. Syst..

[10] Tang L, Xiang Q, Xiang J, Li J (2022). The haplotypes GCA and ACA in ESR1 gene are associated with the susceptibility of recurrent spontaneous abortion (RSA) in Chinese Han: A case-control study and meta-analysis. Medicine.

[11] Körner C, Paulsen J (2004). A world-wide study of high altitude treeline temperatures. J. Biogeogr..

[12] Stephan P, Bramon Mora B, Alexander JM (2021). Positive species interactions shape species' range limits. Oikos.

[13] Afkhami ME, McIntyre PJ, Strauss SY (2014). Mutualist-mediated effects on species' range limits across large geographic scales. Ecol. Lett..

[14] Baer KC, Maron JL (2018). Pre-dispersal seed predation and pollen limitation constrain population growth across the geographic distribution of *Astragalus utahensis*. J. Ecol..

[15] Benning JW, Moeller DA (2019). Maladaptation beyond a geographic range limit driven by antagonistic and mutualistic biotic interactions across an abiotic gradient. Evolution.

[16] He B (2023). A cross-cohort computational framework to trace tumor tissue-of-origin based on RNA sequencing. Sci. Rep..

[17] Terry VC (2004). Biodiversity, distributions and adaptations of arctic species in the context of environmental change. AMBIO A J. Hum. Environ..

[18] Van Der Putten WH (2017). Belowground drivers of plant diversity. Science.

[19] Noffsinger C, Cripps CL (2021). Systematic analysis of Russula in the North American Rocky Mountain alpine zone. Mycologia.

[20] Bardgett RD, van der Putten WH (2014). Belowground biodiversity and ecosystem functioning. Nature.

[21] Dobrovol’skaya TG (2015). The role of microorganisms in the ecological functions of soils. Eurasian Soil Sci..

[22] Smith SE, Read DJ (2010). Mycorrhizal Symbiosis.

[23] Lindahl BD, Tunlid A (2015). Ectomycorrhizal fungi–potential organic matter decomposers, yet not saprotrophs. New Phytol..

[24] Bödeker ITM (2014). Ectomycorrhizal C ortinarius species participate in enzymatic oxidation of humus in northern forest ecosystems. New Phytol..

[25] He B (2020). A neural network framework for predicting the tissue-of-origin of 15 common cancer types based on RNA-Seq data. Front. Bioeng. Biotechnol..

[26] Singh SP (2018). Research on Indian Himalayan Treeline Ecotone: An overview. Trop. Ecol..

[27] Singh SP, Sharma S, Dhyani PP (2019). Himalayan arc and treeline: distribution, climate change responses and ecosystem properties. Biodivers. Conserv..

[28] Dissanayake AJ (2018). Direct comparison of culture-dependent and culture-independent molecular approaches reveal the diversity of fungal endophytic communities in stems of grapevine (*Vitis vinifera*). Fungal Divers..

[29] Vohník M (2020). Ericoid mycorrhizal symbiosis: theoretical background and methods for its comprehensive investigation. Mycorrhiza.

[30] Sun L (2012). Different distribution patterns between putative ercoid mycorrhizal and other fungal assemblages in roots of *Rhododendron decorum* in the Southwest of China. PLoS ONE.

[31] Stehn SE, Webster CR, Jenkins MA, Jose S (2011). High-elevation ground-layer plant community composition across environmental gradients in spruce-fir forests. Ecol. Res..

[32] Verbruggen E, Jansa J, Hammer EC, Rillig MC (2016). Do arbuscular mycorrhizal fungi stabilize litter-derived carbon in soil?. J. Ecol..

[33] Chaurasia B, Pandey A, Palni LMS (2005). Distribution, colonization and diversity of arbuscular mycorrhizal fungi associated with central Himalayan rhododendrons. For. Ecol. Manag..

[34] Freschet GT (2013). Linking litter decomposition of above-and below-ground organs to plant–soil feedbacks worldwide. J. Ecol..

[35] Schroth G, Kolbe D (1994). A method of processing soil core samples for root studies by subsampling. Biol. Fertil. Soils.

[36] Agerer, R. in *Methods in Microbiology* Vol. 23 (eds J. R. Norris, D. J. Read, & A. K. Varma) 25–73 (Academic Press, 1991).

[37] Chapman, H. D. Cation‐exchange capacity. *Methods of soil analysis: Part 2 Chemical and microbiological properties* **9**, 891–901 (1965).

[38] Jackson, M. L. Soil chemical analysis-advanced course. *Soil Chemical Analysis-Advanced Course* (1969).

[39] Phillips JM, Hayman DS (1970). Improved procedures for clearing roots and staining parasitic and vesicular-arbuscular mycorrhizal fungi for rapid assessment of infection. Trans. Br. Mycol. Soc..

[40] Nguyen NH (2016). FUNGuild: An open annotation tool for parsing fungal community datasets by ecological guild. Fungal Ecol..

[41] Goebel BM, Stackebrandt E (1994). Cultural and phylogenetic analysis of mixed microbial populations found in natural and commercial bioleaching environments. Appl. Environ. Microbiol..

[42] Chong J, Liu P, Zhou G, Xia J (2020). Using MicrobiomeAnalyst for comprehensive statistical, functional, and meta-analysis of microbiome data. Nat. Protoc..

[43] Menzel P, Ng KL, Krogh A (2016). Fast and sensitive taxonomic classification for metagenomics with Kaiju. Nat. Commun..

[44] Heberle H, Meirelles GV, da Silva FR, Telles GP, Minghim R (2015). InteractiVenn: A web-based tool for the analysis of sets through Venn diagrams. BMC Bioinform..

[45] Zhang Y (2017). The effects of different human disturbance regimes on root fungal diversity of Rhododendron ovatum in subtropical forests of China. Can. J. For. Res..

[46] Wurzburger N, Higgins BP, Hendrick RL (2012). Ericoid mycorrhizal root fungi and their multicopper oxidases from a temperate forest shrub. Ecol. Evolut..

[47] Abdulraheem MI (2024). Mechanisms of plant epigenetic regulation in response to plant stress: Recent discoveries and implications. Plants.

[48] Vohník M, Albrechtová J (2011). The co-occurrence and morphological continuum between ericoid mycorrhiza and dark septate endophytes in roots of six European rhododendron species. Folia Geobot..

[49] Hashizume Y, Fukuda K, Sahashi N (2010). Effects of summer temperature on fungal endophyte assemblages in Japanese beech (*Fagus crenata*) leaves in pure beech stands. Botany.

[50] Li H-Y (2012). Diversity and cold adaptation of endophytic fungi from five dominant plant species collected from the Baima Snow Mountain, Southwest China. Fungal Divers..

[51] Arnold AE (2007). Understanding the diversity of foliar endophytic fungi: Progress, challenges, and frontiers. Fungal Biol. Rev..

[52] Jiang M (2023). Integrating genomics and metabolomics for the targeted discovery of new cyclopeptides with antifungal activity from a marine-derived fungus *Beauveria felina*. J. Agric. Food Chem..

[53] Christie P, Nicolson TH (1983). Are mycorrhizas absent from the antarctic?. Trans. Br. Mycol. Soc..

[54] Treu R, Laursen GA, Stephenson SL, Landolt JC, Densmore R (1995). Mycorrhizae from Denali national park and preserve, Alaska. Mycorrhiza.

[55] Jumpponen A (2001). Dark septate endophytes—are they mycorrhizal?. Mycorrhiza.

[56] Khan NF, Abdulraheem MI (2023). Trapping alpine mycobiome: Bridging the gap from culture-based isolation to metagenome. Ecol Conserv. Sci.

[57] Lesica P, Antibus RK (1990). The occurrence of mycorrhizae in vascular epiphytes of two Costa Rican rain forests. Biotropica.

[58] Rains KC, Nadkarni NM, Bledsoe CS (2003). Epiphytic and terrestrial mycorrhizas in a lower montane Costa Rican cloud forest. Mycorrhiza.

[59] Bridge P, Spooner B (2001). Soil fungi: Diversity and detection. Plant Soil.

[60] Anderson IC, Cairney JWG (2004). Diversity and ecology of soil fungal communities: Increased understanding through the application of molecular techniques. Environ. Microbiol..

[61] Zhou X, Lu J, Wu B, Guo Z (2022). HOXA11-AS facilitates the proliferation, cell cycle process and migration of keloid fibroblasts through sponging miR-188–5p to regulate VEGFA. J. Dermatol. Sci..

[62] Bougoure DS, Cairney JWG (2005). Fungi associated with hair roots of *Rhododendron lochiae* (Ericaceae) in an Australian tropical cloud forest revealed by culturing and culture-independent molecular methods. Environ. Microbiol..

[63] Koske RE, Gemma JN, Englander L (1990). Vesicular-arbuscular mycorrhizae in Hawaiian Ericales. Am. J. Bot..

[64] Bonfante-Fasolo P (1980). Occurrence of a basidiomycete in living cells of mycorrhizal hair roots of *Calluna vulgaris*. Trans. Br. Mycol. Soc..

[65] Wang Y, Li J, Xiang Q, Tang L (2023). INSR and ISR-1 gene polymorphisms and the susceptibility of essential hypertension: A meta-analysis. Exp. Ther. Med..

[66] Seviour RJ, Willing RR, Chilvers GA (1973). Basidiocarps associated with ericoid mycorrhizas. New Phytol..

[67] Selosse MA (2007). Sebacinales are common mycorrhizal associates of Ericaceae. New Phytol..

[68] Zhang Y, Tang F, Ni J, Dong L, Sun L (2019). Diversity of root-associated fungi of *Rhododendron simsii* in subtropical forests: fungal communities with high resistance to anthropogenic disturbances. J. For. Res..

[69] Ping Y (2016). Vertical zonation of soil fungal community structure in a Korean pine forest on Changbai Mountain, China. World J. Microbiol. Biotechnol..

[70] Shen C (2014). Contrasting elevational diversity patterns between eukaryotic soil microbes and plants. Ecology.

[71] Ni Y, Yang T, Zhang K, Shen C, Chu H (2018). Fungal communities along a small-scale elevational gradient in an alpine tundra are determined by soil carbon nitrogen ratios. Front. Microbiol..

[72] Tian C (2010). Regulation of the nitrogen transfer pathway in the arbuscular mycorrhizal symbiosis: Gene characterization and the coordination of expression with nitrogen flux. Plant Physiol..

[73] Leopold DR (2016). Ericoid fungal diversity: Challenges and opportunities for mycorrhizal research. Fungal Ecol..

[74] Ahmed S (2022). Molecular communication network and its applications in crop sciences. Planta.

[75] Fehrer J, Réblová M, Bambasová V, Vohník M (2019). The root-symbiotic *Rhizoscyphus ericae* aggregate and Hyaloscypha (Leotiomycetes) are congeneric: Phylogenetic and experimental evidence. Stud. Mycol..

[76] Foster ZSL, Weiland JE, Scagel CF, Grünwald NJ (2020). The composition of the fungal and oomycete microbiome of rhododendron roots under varying growth conditions, nurseries, and cultivars. Phytobiomes J..

[77] Timling I, Walker DA, Nusbaum C, Lennon NJ, Taylor DL (2014). Rich and cold: diversity, distribution and drivers of fungal communities in patterned-ground ecosystems of the North American Arctic. Mol. Ecol..

[78] McCarthy-Neumann S, Ibáñez I (2012). Tree range expansion may be enhanced by escape from negative plant–soil feedbacks. Ecology.

[79] Keymer DP, Lankau RA (2017). Disruption of plant–soil–microbial relationships influences plant growth. J. Ecol..

[80] Peay KG (2016). The mutualistic niche: mycorrhizal symbiosis and community dynamics. Annu. Rev. Ecol. Evol. Syst..

[81] Van Wees SCM, Van der Ent S, Pieterse CMJ (2008). Plant immune responses triggered by beneficial microbes. Curr. Opin. Plant Biol..

[82] Walitang DI (2017). Characterizing endophytic competence and plant growth promotion of bacterial endophytes inhabiting the seed endosphere of Rice. BMC Microbiol..

