# Supplementary material for: Deciphering mycobiota and its functional dynamics in root hairs of Rhododendron campanulatum D. Don through Next-gen sequencing
Source: Sci Rep. 2024 May 4;14:10294. doi: 10.1038/s41598-024-61120-4 (PMC11069570; doi:10.1038/s41598-024-61120-4)
Supplement: Supplementary file 1 — Supplementary Information. [file 41598_2024_61120_MOESM1_ESM.docx]

**Deciphering mycobiota and its functional dynamics in root hairs of *Rhododendron campanulatum* D. Don through Next-gen Sequencing**

**Nafeesa Farooq Khan*^1^, Sheikh Sajad Ahmed^1^, Mukhtar Iderawumi Abdulraheem^2,3^, Zafar Ahmad Reshi ^1^, Abdul Wahab^4,5^, Gholaremza Abdi^6**^**

1. Biological Invasion Lab, Department of Botany, University of Kashmir, Srinagar-190006, Jammu & Kashmir, India (NFK: [khannafeesacsiriiim@gmail.com](mailto:khannafeesacsiriiim@gmail.com)) (SSA: [sheikhsajad.scholar@kashmiruniversity.net](mailto:sheikhsajad.scholar@kashmiruniversity.net) ) (ZAR: [zreshii@gmail.com](mailto:zreshii@gmail.com))
2. Department of Electrical Engineering, Henan Agricultural University, Zhengzhou 450002, China. [abdulraheem@stu.henau.edu.cn](mailto:abdulraheem@stu.henau.edu.cn)
3. Henan International Joint Laboratory of Laser Technology in Agriculture Science, Zhengzhou 450002, China
4. University of Chinese Academy of Sciences, Beijing 100049, China. [wahabcrop_science@mails.ucas.ac.cn](mailto:Wahabcrop_science@mail.ucas.ac.cn)
5. Shanghai Center for Plant Stress Biology, CAS Centre for Excellence in Molecular Plant Sciences, Chinese Academy of Sciences, Shanghai 200032, China
6. Department of Biotechnology, Persian Gul Research Institute, Persian Gulf University, Bushehr, 75169, Iran. [abdi@pgu.ac.ir](mailto:abdi@pgu.ac.ir)

*Email: **[abdi@pgu.ac.ir](mailto:abdi@pgu.ac.ir); *[khannafeesacsiriiim@gmail.com](mailto:khannafeesacsiriiim@gmail.com)

Supplementary Table 1: Soil characteristics at the two study sites.

| *Rhododendron campanulatum* | | | | | | | | | |
| --- | --- | --- | --- | --- | --- | --- | --- | --- | --- |
| Site | Altitude (m.a.s.l) | OC  (%) | EC | pH | N (ppm) | P  (ppm) | K (ppm) | Mg  (ppm) | Ca  (ppm) |
| Sinthan Top | 3396 | 2.46 | 0.08 | 5.8 | 200.89 | 8.03 | 87.9 | 119.1 | 779.01 |
|  | 3552 | 2.1 | 0.1 | 6 | 171.8 | 5.35 | 56.2 | 115.1 | 778.1 |
| Apharwat | 3610 | 0.66 | 0.06 | 5.93 | 58.03 | 4.01 | 80.35 | 109.8 | 763.3 |
|  | 3800 | 0.62 | 0.05 | 5.5 | 59.3 | 4.46 | 81.2 | 111.1 | 762.5 |


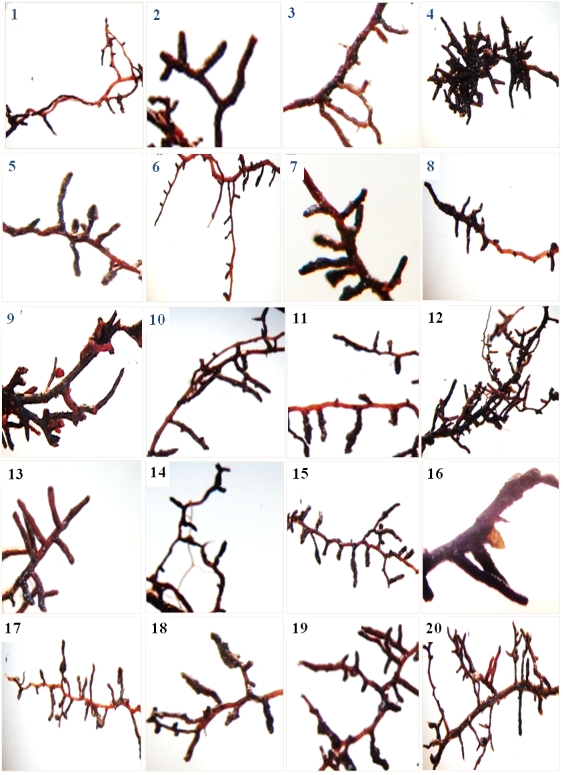


Supplementary Figure S1: Root morphotypes identified in *R. campanulatum*

Supplementary Table S2: Taxonomic description of root morphotypes

| S.No | Description | Morphotypes |
| --- | --- | --- |
| 1 | Orange-brown, Thin long hyphal network | Irregular |
| 2 | Dark brown, uniformly thick dichotomous branching | Dichotomous |
| 3 | Light Brown, fibrous root tips, irregular mycorrhizal structure | Not branched |
| 4 | Black, several short dichotomously branched mycorrhizae enveloped together with emanating hyphae | Coralloid |
| 5 | Orange-tan colour round thick ends paler ends | Monopodial pinnate |
| 6 | Reddish-brown, small infected root tips | Dichotomous |
| 7 | Brownish/black, arranged in more than three rows | Monopodial pyramidal |
| 8 | Black and tan, parallel branching | Monopodial pinnate |
| 9 | Shiny reddish-brown mycorrhizal tips solitary attached to the main axis, apices broad and base narrow | Other morphotypes |
| 10 | Black reddish tips, densely packed with more short branches | Dichotomous |
| 11 | Yellow Reddish, slender and short tips | Monopodial pinnate |
| 12 | Orange, long branched shiny hyphal structure | Coralloid |
| 13 | Whitish, pointed tips wider base attached to the main axis | Monopodial pinnate |
| 14 | Dark Brown /Reddish, short branches | Tortuous |
| 15 | Brown, tips bifurcated | Not branched |
| 16 | Black/Yellowish | sessile |
| 17 | Whitish, clustered root tips, tips grow conjointly | Monopodial pyramidal |
| 18 | Dark brown to light brown, several curves, no plane of mycorrhizal tips thin radiating hyphae, tips tapering | Irregular |
| 19 | Deep red colour | Monopodial pyramidal |
| 20 | Reddish Brown, pale tips without main axis | Monopodial pinnate |

| **Supplementary Table S3:** Numbers of reads allocated to each identified taxon from each sample | | | | | | | | | | | | | | | | | | | | | | | | | | | | | | |  |
| --- | --- | --- | --- | --- | --- | --- | --- | --- | --- | --- | --- | --- | --- | --- | --- | --- | --- | --- | --- | --- | --- | --- | --- | --- | --- | --- | --- | --- | --- | --- | --- |
| **Samples** | | | | | | | | | | | | | | | | | | | | | | | | | | | | | | |  |
| **A1** | | | **A2** | | **A3** | | | **D1** | | | **D2** | | **C2** | | | | **C3** | | | **E1** | | | **E2** | | | **E3** | | | **D3/C1** | |  |
| **Taxa** | | **Cumulative Reads** | **Taxa** | **Cumulative Reads** | **Taxa** | **Cumulative Reads** | **Taxa** | | **Cumulative Reads** | **Taxa** | | **Cumulative Reads** | | **Taxa** | **Cumulative Reads** | **Taxa** | | **Cumulative Reads** | **Taxa** | | **Cumulative Reads** | **Taxa** | | **Cumulative Reads** | **Taxa** | | **Cumulative Reads** | **UNKNOWN READS** | |  |  |
| *Lobosporangium transversale* | | **565** | *Lobosporangium transversale* | **126** | *Lobosporangium transversale* | **645** | *Albifimbria verrucaria* | | **2** | *Agaricus bisporus* | | **9** | | *Lobosporangium transversale* | **2** | *Agaricus bisporus* | | **8** | *Alternaria solani* | | **2** | *Aspergillus penicillioides* | | **73** | *Albifimbria verrucaria* | | **1** |  | |  |  |
| *Malassezia pachydermatis* | | **49** | *Schizosaccharomyces octosporus* | **120** | *Schizosaccharomyces octosporus* | **179** | *Alternaria alternata* | | **1** | *Laccaria bicolor* | | **7** | | *Fusarium graminearum* | **1** | *Laccaria bicolor* | | **1** | *Botryosphaeria dothidea* | | **1** | *Aspergillus westerdijkiae* | | **1** | *Alternaria alternata* | | **1** |  | |  |  |
| *Melampsora larici-populina* | | **48** | *Paracoccidioides brasiliensis* | **87** | *Paracoccidioides brasiliensis* | **102** | *Alternaria solani* | | **5** | *Phanerochaete carnosa* | | **5** | | *Postia placenta* | **1** | *Serpula lacrymans* | | **1** | *Truncatella angustata* | | **2** | *Bartalinia robillardoides* | | **1** | *Alternaria solani* | | **17** |  | |  |  |
| *Schizosaccharomyces octosporus* | | **45** | *Agaricus bisporus* | **72** | *Rhodotorula graminis* | **73** | *Botryosphaeria dothidea* | | **3** | *Penicillium arizonense* | | **2** | | *Phanerochaete carnosa* | **1** | *Punctularia strigosozonata* | | **1** | *[Candida] pseudoaaseri* | | **1** | *Brunaudia phormigena* | | **1** | *Aspergillus sp. 06 SMR-2010* | | **1** |  | |  |  |
| *Rhodotorula graminis* | | **33** | *Rhodotorula graminis* | **42** | *Punctularia strigosozonata* | **55** | *Brunaudia phormigena* | | **1** | *Histoplasma capsulatum* | | **1** | |  |  | *Phanerochaete carnosa* | | **1** | *Acidea extrema* | | **6** | *Chlorociboria aeruginosa* | | **1** | *Fusarium acuminatum* | | **2** |  | |  |  |
| *Eutypa lata* | | **31** | *Punctularia strigosozonata* | **26** | *Eutypa lata* | **40** | *Chalara hyalocuspica* | | **1** | *Paracoccidioides brasiliensis* | | **1** | |  |  | *Rhodotorula graminis* | | **1** | *Allantophomopsis lycopodina* | | **2** | *Ciliciopodium hyalinum* | | **2** | *Fusarium lateritium* | | **1** |  | |  |  |
| *Kockovaella imperatae* | | **28** | *Tremella mesenterica* | **25** | *Pichia kudriavzevii* | **25** | *Claussenomyces sp. PDD 55517* | | **1** | *Colletotrichum graminicola* | | **1** | |  |  | *Trichophyton rubrum* | | **1** | *Bartalinia pondoensis* | | **1** | *Cladophialophora chaetospira* | | **12** | *Gyoerffyella sp. RJ-2015* | | **1** |  | |  |  |
| *Paracoccidioides brasiliensis* | | **23** | *Pichia kudriavzevii* | **19** | *Tremella mesenterica* | **25** | *Fusarium lateritium* | | **2** | *Clavispora lusitaniae* | | **1** | |  |  | *Neofusicoccum parvum* | | **1** | *Bartalinia robillardoides* | | **1** | *Cladophialophora sp. V475* | | **1** | *Ilyonectria robusta* | | **25** |  | |  |  |
| *Trichoderma atroviride* | | **21** | *Melampsora larici-populina* | **19** | *Agaricus bisporus* | **23** | *Ilyonectria robusta* | | **49** | *Ogataea polymorpha* | | **1** | |  |  | *Leptosphaeria maculans* | | **1** | *Boeremia exigua* | | **1** | *Cladosporium dominicanum* | | **2** | *Truncatella angustata* | | **1** |  | |  |  |
| *Tuber melanosporum* | | **16** | *Trichosporon asahii* | **16** | *Clavispora lusitaniae* | **22** | *Phoma herbarum* | | **1** | *Kwoniella pini* | | **1** | |  |  | *Colletotrichum orchidophilum* | | **1** | *Brunaudia phormigena* | | **7** | *Cladosporium sp. SS-S12* | | **4** | *Acidea extrema* | | **9** |  | |  |  |
| *Saitoella complicata* | | **15** | *Pneumocystis jirovecii* | **14** | *Leptosphaeria maculans* | **21** | *Truncatella angustata* | | **4** | *Rhodotorula graminis* | | **1** | |  |  | *Ascoidea rubescens* | | **1** | *Cadophora malorum* | | **1** | *Cladosporium tenuissimum* | | **2** | *Alternaria citri* | | **1** |  | |  |  |
| *Diplodia corticola* | | **15** | *Ogataea polymorpha* | **14** | *Pneumocystis jirovecii* | **20** | *Acidea extrema* | | **14** | *Malassezia pachydermatis* | | **1** | |  |  | *Meyerozyma guilliermondii* | | **1** | *Cadophora sp.* | | **2** | *Claussenomyces sp. PDD 55517* | | **3** | *Ascochyta phacae* | | **4** |  | |  |  |
| *Botrytis cinerea* | | **13** | *Dichomitus squalens* | **13** | *Paracoccidioides lutzii* | **20** | *Acremonium cf. curvulum CBS 100551* | | **1** | *Lobosporangium transversale* | | **1** | |  |  | *Saccharomyces cerevisiae* | | **1** | *Calycina marina* | | **1** | *Collophora paarla* | | **1** | *Aspergillus niger* | | **1** |  | |  |  |
| *Marssonina brunnea* | | **12** | *Kockovaella imperatae* | **13** | *Melampsora larici-populina* | **19** | *Acremonium sp. r091* | | **2** |  | |  | |  |  |  | |  | *Ciliciopodium hyalinum* | | **4** | *Crucellisporium umtamvunae* | | **1** | *Bartalinia pondoensis* | | **2** |  | |  |  |
| *Metschnikowia bicuspidata* | | **12** | *Saitoella complicata* | **12** | *Kockovaella imperatae* | **18** | *Allantophomopsis lycopodina* | | **2** |  | |  | |  |  |  | |  | *Cladosporium tenuissimum* | | **1** | *Darkera parca* | | **1** | *Bartalinia robillardoides* | | **7** |  | |  |  |
| *Agaricus bisporus* | | **11** | *Endocarpon pusillum* | **11** | *Fonsecaea erecta* | **16** | *Alternaria alternata* | | **1** |  | |  | |  |  |  | |  | *Claussenomyces sp. PDD 55517* | | **1** | *Eleutheromyces subulatus* | | **1** | *Boeremia exigua* | | **9** |  | |  |  |
| *Mixia osmundae* | | **10** | *Clavispora lusitaniae* | **10** | *Schizosaccharomyces japonicus* | **14** | *Articulospora tetracladia* | | **2** |  | |  | |  |  |  | |  | *Claussenomyces sp. PDD 80575* | | **1** | *Fusarium sp.* | | **1** | *Brunaudia phormigena* | | **21** |  | |  |  |
| *Glarea lozoyensis* | | **8** | *Fibroporia radiculosa* | **9** | *Ogataea polymorpha* | **10** | *Ascochyta phacae* | | **3** |  | |  | |  |  |  | |  | *Clavispora lusitaniae* | | **1** | *Gyoerffyella rotula* | | **1** | *Calloria urticae* | | **1** |  | |  |  |
| *Colletotrichum higginsianum* | | **7** | *Laccaria bicolor* | **9** | *Kluyveromyces lactis* | **10** | *Bartalinia pondoensis* | | **13** |  | |  | |  |  |  | |  | *Crocicreas sp. MUT 4416* | | **7** | *Gyoerffyella sp. B54J5* | | **1** | *Camarosporium brabeji* | | **2** |  | |  |  |
| *Metarhizium brunneum* | | **7** | *Eutypa lata* | **8** | *Colletotrichum higginsianum* | **9** | *Bartalinia robillardoides* | | **29** |  | |  | |  |  |  | |  | *Crucellisporium umtamvunae* | | **1** | *Gyoerffyella sp. RJ-2015* | | **7** | *Chaetomium sp. YJM-2013* | | **1** |  | |  |  |
| *Sphaerulina musiva* | | **7** | *Fonsecaea erecta* | **7** | *Sugiyamaella lignohabitans* | **7** | *Boeremia exigua* | | **6** |  | |  | |  |  |  | |  | *Darkera picea* | | **1** | *Humicola grisea* | | **1** | *Ciliciopodium brevipes* | | **1** |  | |  |  |
| *Punctularia strigosozonata* | | **7** | *Leptosphaeria maculans* | **7** | *Kwoniella mangrovensis* | **7** | *Brunaudia phormigena* | | **34** |  | |  | |  |  |  | |  | *Didymella glomerata* | | **2** | *Hyphodiscus sp. UBCF23770* | | **3** | *Ciliciopodium hyalinum* | | **2** |  | |  |  |
| *Pochonia chlamydosporia* | | **6** | *Zymoseptoria tritici* | **6** | *Batrachochytrium dendrobatidis* | **7** | *Cadophora luteo-olivacea* | | **1** |  | |  | |  |  |  | |  | *Entrophospora sp. JJ61* | | **1** | *Inocybe cf. friesii SMI337* | | **1** | *Cistella grevillei* | | **3** |  | |  |  |
| *Wallemia ichthyophaga* | | **6** | *Paracoccidioides lutzii* | **5** | *Saitoella complicata* | **6** | *Cadophora malorum* | | **1** |  | |  | |  |  |  | |  | *Fusarium lateritium* | | **1** | *Inocybe fuscidula* | | **2** | *Cladophialophora chaetospira* | | **1** |  | |  |  |
| *Leptosphaeria maculans* | | **5** | *Tuber melanosporum* | **5** | *Coniophora puteana* | **6** | *Cadophora orchidicola* | | **2** |  | |  | |  |  |  | |  | *Fusarium sp. HMA-16* | | **2** | *Inocybe lanuginosa* | | **2** | *Cladosporium delicatulum* | | **1** |  | |  |  |
| *Coniophora puteana* | | **5** | *Serpula lacrymans* | **5** | *Trichosporon asahii* | **6** | *Cadophora sp.* | | **3** |  | |  | |  |  |  | |  | *Fusarium sp. SS-R6* | | **2** | *Isaria tenuipes* | | **3** | *Claussenomyces sp. PDD 55517* | | **1** |  | |  |  |
| *Fusarium oxysporum* | | **4** | *Batrachochytrium dendrobatidis* | **5** | *Tuber melanosporum* | **5** | *Calonectria pentaseptata* | | **1** |  | |  | |  |  |  | |  | *Geomyces sp. WNF-15A* | | **2** | *Leohumicola verrucosa* | | **1** | *Claussenomyces sp. PDD 80575* | | **1** |  | |  |  |
| *Fusarium graminearum* | | **4** | *Schizosaccharomyces japonicus* | **4** | *Phycomyces blakesleeanus* | **5** | *Ciliciopodium hyalinum* | | **17** |  | |  | |  |  |  | |  | *Glonium pusillum* | | **1** | *Leptodontidium elatius* | | **3** | *Clavulina cinerea* | | **1** |  | |  |  |
| *Chaetomium globosum* | | **4** | *Fonsecaea pedrosoi* | **4** | *Pneumocystis murina* | **4** | *Cistella grevillei* | | **4** |  | |  | |  |  |  | |  | *Gyoerffyella sp. B54A4* | | **2** | *Meyerozyma caribbica* | | **9** | *Coleophoma eucalyptorum* | | **1** |  | |  |  |
| *Parastagonospora nodorum* | | **4** | *Trametes versicolor* | **4** | *Wallemia ichthyophaga* | **4** | *Cistella sp. EF-8* | | **1** |  | |  | |  |  |  | |  | *Hebeloma aff. remyi UBC F23896* | | **1** | *Mortierella minutissima* | | **1** | *Cortinarius sp. PDD 97558* | | **1** |  | |  |  |
| *Naumovozyma dairenensis* | | **4** | *Coniophora puteana* | **4** | *Mixia osmundae* | **4** | *Cladophialophora chaetospira* | | **2** |  | |  | |  |  |  | |  | *Hyalodendriella betulae* | | **2** | *Mortierella sp. F121* | | **2** | *Crocicreas sp. MUT 4416* | | **21** |  | |  |  |
| *Tremella mesenterica* | | **4** | *Wallemia ichthyophaga* | **4** | *Trichoderma atroviride* | **3** | *Cladosporium allicinum* | | **2** |  | |  | |  |  |  | |  | *Hymenoscyphus fructigenus* | | **1** | *Mortierella sp. WD25F* | | **3** | *Crucellisporium umtamvunae* | | **2** |  | |  |  |
| *Trichoderma virens* | | **3** | *Mixia osmundae* | **4** | *Scedosporium apiospermum* | **3** | *Cladosporium delicatulum* | | **10** |  | |  | |  |  |  | |  | *Hymenoscyphus kiko* | | **1** | *Mycena purpureofusca* | | **1** | *Cudoniella clavus* | | **4** |  | |  |  |
| *Phialocephala scopiformis* | | **3** | *Phycomyces blakesleeanus* | **4** | *Sporothrix schenckii* | **3** | *Cladosporium langeronii* | | **1** |  | |  | |  |  |  | |  | *Hymenoscyphus ohakune* | | **8** | *Mycocentrospora acerina* | | **2** | *Dactylonectria torresensis* | | **12** |  | |  |  |
| *Pseudogymnoascus destructans* | | **3** | *Metschnikowia bicuspidata* | **3** | *Talaromyces stipitatus* | **3** | *Cladosporium tenuissimum* | | **1** |  | |  | |  |  |  | |  | *Fusarium sp. SS-R6* | | **2** | *Mycocentrospora cantuariensis* | | **1** | *Didymella glomerata* | | **4** |  | |  |  |
| *Pseudocercospora fijiensis* | | **3** | *Kluyveromyces lactis* | **3** | *Metarhizium brunneum* | **2** | *Claussenomyces sp. PDD 55517* | | **10** |  | |  | |  |  |  | |  | *Hymenoscyphus scutula* | | **3** | *Mycosymbioces mycenaphila* | | **2** | *Entrophospora sp. JJ61* | | **2** |  | |  |  |
| *Clavispora lusitaniae* | | **3** | *Kwoniella mangrovensis* | **3** | *Botrytis cinerea* | **2** | *Coccinonectria pachysandricola* | | **1** |  | |  | |  |  |  | |  | *Inocybe auricoma* | | **1** | *Nectria pseudotrichia* | | **1** | *Fusarium avenaceum* | | **2** |  | |  |  |
| *Pichia kudriavzevii* | | **3** | *Pneumocystis murina* | **2** | *Coccidioides posadasii* | **2** | *Coleophoma eucalyptorum* | | **1** |  | |  | |  |  |  | |  | *Inocybe cf. friesii SMI337* | | **1** | *Nectria sp.* | | **1** | *Fusarium equiseti* | | **2** |  | |  |  |
| *Postia placenta* | | **3** | *Schizosaccharomyces pombe* | **2** | *Parastagonospora nodorum* | **2** | *Collembolispora barbata* | | **1** |  | |  | |  |  |  | |  | *Inocybe rimosa* | | **1** | *Oidiodendron maius* | | **1** | *Fusarium lateritium* | | **11** |  | |  |  |
| *Schizosaccharomyces japonicus* | | **2** | *Parastagonospora nodorum* | **2** | *Pneumocystis carinii* | **1** | *Colletotrichum gloeosporioides* | | **1** |  | |  | |  |  |  | |  | *Isaria tenuipes* | | **1** | *Ophiocordyceps sinensis* | | **6** | *Fusarium merismoides* | | **1** |  | |  |  |
| *Pneumocystis jirovecii* | | **2** | *Setosphaeria turcica* | **2** | *Pestalotiopsis fici* | **1** | *Cordyceps brongniartii* | | **1** |  | |  | |  |  |  | |  | *Laccaria sp. HKAS 53701* | | **1** | *Papiliotrema pseudoalba* | | **3** | *Fusarium sp.* | | **2** |  | |  |  |
| *Fonsecaea erecta* | | **2** | *Verticillium dahliae* | **2** | *Colletotrichum graminicola* | **1** | *Crocicreas sp. MUT 4416* | | **25** |  | |  | |  |  |  | |  | *Leohumicola minima* | | **2** | *Paracremonium inflatum* | | **19** | *Fusarium sp. CBS 119214* | | **5** |  | |  |  |
| *Phialophora attae* | | **2** | *Trichoderma atroviride* | **2** | *Verticillium dahliae* | **1** | *Cudoniella clavus* | | **4** |  | |  | |  |  |  | |  | *Leohumicola sp. DAOM 230084* | | **1** | *Penicillium chloroleucon* | | **1** | *Fusarium sp. FTSC_grp1 KOD-2010* | | **3** |  | |  |  |
| *Colletotrichum graminicola* | | **2** | *Pestalotiopsis fici* | **2** | *Trichoderma virens* | **1** | *Cylindrodendrum hubeiense* | | **1** |  | |  | |  |  |  | |  | *Leptosphaeria sclerotioides* | | **1** | *Pezicula ericae* | | **2** | *Fusarium sp. SS-R6* | | **1** |  | |  |  |
| *Verticillium dahliae* | | **2** | *Fomitiporia mediterranea* | **2** | *Fusarium graminearum* | **1** | *Dactylonectria torresensis* | | **8** |  | |  | |  |  |  | |  | *Lunulospora curvula* | | **1** | *Pezicula sp. 4 CC-2015* | | **1** | *Fusicolla matuoi* | | **4** |  | |  |  |
| *Sporothrix schenckii* | | **2** | *Postia placenta* | **2** | *Phaeoacremonium minimum* | **1** | *Didymella glomerata* | | **1** |  | |  | |  |  |  | |  | *Microsphaeropsis proteae* | | **2** | *Phacidium grevilleae* | | **6** | *Glonium pusillum* | | **3** |  | |  |  |
| *Phaeoacremonium minimum* | | **2** | *Malassezia pachydermatis* | **2** | *Phialocephala scopiformis* | **1** | *Entrophospora sp. JJ61* | | **3** |  | |  | |  |  |  | |  | *Monilinia laxa* | | **3** | *Phialea strobilina* | | **23** | *Gyoerffyella rotula* | | **2** |  | |  |  |
| *Neofusicoccum parvum* | | **2** | *Spizellomyces punctatus* | **2** | *Pseudogymnoascus destructans* | **1** | *Fulvoflamma eucalypti* | | **6** |  | |  | |  |  |  | |  | *Mortierella sp. WD25F* | | **1** | *Phlyctema vagabunda* | | **14** | *Gyoerffyella sp. B54A4* | | **2** |  | |  |  |
| *Ogataea polymorpha* | | **2** | *Pneumocystis carinii* | **1** | *Blastomyces gilchristii* | **1** | *Fusarium lateritium* | | **26** |  | |  | |  |  |  | |  | *Mycochaetophora gentianae* | | **1** | *Scleropezicula alnicola* | | **1** | *Gyoerffyella sp. B54J5* | | **4** |  | |  |  |
| *Heterobasidion irregulare* | | **2** | *Coccidioides posadasii* | **1** | *Uncinocarpus reesii* | **1** | *Fusarium sp.* | | **3** |  | |  | |  |  |  | |  | *Mycosymbioces mycenaphila* | | **6** | *Serendipita vermifera* | | **2** | *Haplographium catenatum* | | **1** |  | |  |  |
| *Serpula lacrymans* | | **2** | *Blastomyces gilchristii* | **1** | *Fonsecaea pedrosoi* | **1** | *Fusarium sp. C1* | | **1** |  | |  | |  |  |  | |  | *Neofabraea krawtzewii* | | **2** | *Thuemenidium atropurpureum* | | **3** | *Hyalodendriella betulae* | | **3** |  | |  |  |
| *Spizellomyces punctatus* | | **2** | *Talaromyces stipitatus* | **1** | *Phialophora attae* | **1** | *Fusarium sp. CBS 119214* | | **5** |  | |  | |  |  |  | |  | *Neonectria punicea* | | **1** | *Thyronectria berolinensis* | | **1** | *Hymenoscyphus caudatus* | | **1** |  | |  |  |
| *Pneumocystis murina* | | **1** | *Capronia epimyces* | **1** | *Endocarpon pusillum* | **1** | *Fusarium sp. FTSC_grp1 KOD-2010* | | **1** |  | |  | |  |  |  | |  | *Ophiocordyceps crassispora* | | **2** | *Thyronectria sinopica* | | **3** | *Hymenoscyphus fructigenus* | | **2** |  | |  |  |
| *Paracoccidioides lutzii* | | **1** | *Exophiala xenobiotica* | **1** | *Metschnikowia bicuspidata* | **1** | *Fusarium sp. HMA-14* | | **1** |  | |  | |  |  |  | |  | *Ophiocordyceps sinensis* | | **1** | *Tolypocladium inflatum* | | **2** | *Hymenoscyphus ohakune* | | **14** |  | |  |  |
| *Trichophyton verrucosum* | | **1** | *Sphaerulina musiva* | **1** | *Vanderwaltozyma polyspora* | **1** | *Fusarium sp. ICMP 19978* | | **1** |  | |  | |  |  |  | |  | *Papiliotrema pseudoalba* | | **1** | *Trichoderma asperellum* | | **3** | *Hymenoscyphus scutula* | | **5** |  | |  |  |
| *Penicillium digitatum* | | **1** | *Bipolaris maydis* | **1** | *Kuraishia capsulata* | **1** | *Fusarium sp. SS-R6* | | **6** |  | |  | |  |  |  | |  | *Pezicula cinnamomea* | | **1** | *Trichoderma paraviridescens* | | **12** | *Hyphodiscus sp. UBCF23770* | | **1** |  | |  |  |
| *Fonsecaea pedrosoi* | | **1** | *Botrytis cinerea* | **1** | *Fomitiporia mediterranea* | **1** | *Fusarium tricinctum* | | **1** |  | |  | |  |  |  | |  | *Pezicula ericae* | | **2** | *Trichoderma pubescens* | | **1** | *Hypoxylon monticulosum* | | **1** |  | |  |  |
| *Endocarpon pusillum* | | **1** | *Pseudogymnoascus destructans* | **1** | *Postia placenta* | **1** | *Fusicolla acetilerea* | | **1** |  | |  | |  |  |  | |  | *Phacidiopycnis washingtonensis* | | **3** | *Trichoderma viride* | | **1** | *Ilyonectria leucospermi* | | **10** |  | |  |  |
| *Fusarium verticillioides* | | **1** | *Colletotrichum higginsianum* | **1** | *Heterobasidion irregulare* | **1** | *Fusicolla matuoi* | | **2** |  | |  | |  |  |  | |  | *Phacidium grevilleae* | | **15** | *Truncatella angustata* | | **2** | *Ilyonectria sp. 2 AC-2011* | | **6** |  | |  |  |
| *Scedosporium apiospermum* | | **1** | *Verticillium alfalfae* | **1** | *Laccaria bicolor* | **1** | *Geomyces sp. WNF-15A* | | **3** |  | |  | |  |  |  | |  | *Phialocephala virens* | | **1** | *Volutella rosea* | | **4** | *Lachnum virgineum* | | **7** |  | |  |  |
| *Magnaporthe oryzae* | | **1** | *Cordyceps militaris* | **1** | *Serpula lacrymans* | **1** | *Glonium pusillum* | | **1** |  | |  | |  |  |  | |  | *Phlyctema vagabunda* | | **7** | *Xenopolyscytalum pinea* | | **1** | *Laetinaevia carneoflavida* | | **1** |  | |  |  |
| *Grosmannia clavigera* | | **1** | *Sporothrix schenckii* | **1** | *Mitosporidium daphniae* | **1** | *Gymnostellatospora sp. SL0219* | | **1** |  | |  | |  |  |  | |  | *Phoma herbarum* | | **1** | *Alternaria solani* | | **1** | *Lambertella pruni* | | **1** |  | |  |  |
| *Zymoseptoria tritici* | | **1** | *Dactylellina haptotyla* | **1** |  |  | *Gyoerffyella sp. B54A4* | | **5** |  | |  | |  |  |  | |  | *Pilidium concavum* | | **1** | *Fusarium fujikuroi* | | **1** | *Lecythophora sp. UBCtra1453C* | | **1** |  | |  |  |
| *Bipolaris sorokiniana* | | **1** | *[Candida] auris* | **1** |  |  | *Haplographium catenatum* | | **3** |  | |  | |  |  |  | |  | *Pseudogymnoascus sp. FI687* | | **1** | *Gyoerffyella sp. RJ-2015* | | **1** | *Leohumicola minima* | | **1** |  | |  |  |
| *Dactylellina haptotyla* | | **1** | *Zygosaccharomyces rouxii* | **1** |  |  | *Humicola grisea* | | **1** |  | |  | |  |  |  | |  | *Sarimanas shirakamiense* | | **2** | *Ilyonectria robusta* | | **4** | *Leohumicola sp. DAOM 230084* | | **1** |  | |  |  |
| *Meyerozyma guilliermondii* | | **1** | *Kuraishia capsulata* | **1** |  |  | *Hyalodendriella betulae* | | **5** |  | |  | |  |  |  | |  | *Spirosphaera floriformis* | | **8** | *Penicillium allii* | | **31** | *Leptosphaerulina australis* | | **1** |  | |  |  |
| *[Candida] auris* | | **1** | *Sugiyamaella lignohabitans* | **1** |  |  | *Hymenoscyphus ohakune* | | **21** |  | |  | |  |  |  | |  | *Stagonosporopsis cucurbitacearum* | | **2** | *Tolypocladium inflatum* | | **1** | *Meliniomyces bicolor* | | **6** |  | |  |  |
| *Tetrapisispora blattae* | | **1** | *Phanerochaete carnosa* | **1** |  |  | *Hymenoscyphus scutula* | | **12** |  | |  | |  |  |  | |  | *Thelebolus sp. MUT 5300* | | **4** | *Trichoderma viride* | | **9** | *Meliniomyces variabilis* | | **1** |  | |  |  |
| *Tetrapisispora phaffii* | | **1** | *Schizophyllum commune* | **1** |  |  | *Hymenoscyphus sp. AL15m6* | | **1** |  | |  | |  |  |  | |  | *Thuemenidium atropurpureum* | | **6** |  | |  | *Metarhizium carneum* | | **1** |  | |  |  |
| *Sugiyamaella lignohabitans* | | **1** | *Cryptococcus neoformans* | **1** |  |  | *Ilyonectria capensis* | | **1** |  | |  | |  |  |  | |  | *Tolypocladium inflatum* | | **1** |  | |  | *Microsphaeropsis proteae* | | **9** |  | |  |  |
| *Fomitiporia mediterranea* | | **1** |  |  |  |  | *Ilyonectria destructans* | | **1** |  | |  | |  |  |  | |  | *Torrendiella eucalypti* | | **1** |  | |  | *Mollisia dextrinospora* | | **1** |  | |  |  |
| *Phanerochaete carnosa* | | **1** |  |  |  |  | *Ilyonectria leucospermi* | | **8** |  | |  | |  |  |  | |  | *Truncatella angustata* | | **29** |  | |  | *Monilinia fructicola* | | **1** |  | |  |  |
| *Mitosporidium daphniae* | | **1** |  |  |  |  | *Ilyonectria liriodendri* | | **1** |  | |  | |  |  |  | |  | *Verrucoconiothyrium nitidae* | | **1** |  | |  | *Monodictys arctica* | | **3** |  | |  |  |
|  | |  |  |  |  |  | *Ilyonectria protearum* | | **2** |  | |  | |  |  |  | |  | *Vestigium trifidum* | | **6** |  | |  | *Mycena sp. MLB-2010a* | | **1** |  | |  |  |
|  | |  |  |  |  |  | *Ilyonectria rufa* | | **1** |  | |  | |  |  |  | |  | *Vibrissea sp. PDD 99892* | | **1** |  | |  | *Mycosymbioces mycenaphila* | | **4** |  | |  |  |
|  |  | |  |  |  |  | *Lachnellula tricolor* | | **3** |  | |  | |  |  |  | |  | *Vibrissea truncorum* | | **2** |  | |  | *Nectria pseudotrichia* | | **1** |  | |  | |
|  | |  |  |  |  |  | *Lachnum virgineum* | | **2** |  | |  | |  |  |  | |  | *Xenostigmina zilleri* | | **1** |  | |  | *Neofabraea perennans* | | **1** |  | |  |  |
|  | |  |  |  |  |  | *Laetinaevia carneoflavida* | | **2** |  | |  | |  |  |  | |  | *Pilidium concavum* | | **1** |  | |  | *Neonectria lugdunensis* | | **5** |  | |  |  |
|  | |  |  |  |  |  | *Lauriomyces bellulus* | | **1** |  | |  | |  |  |  | |  |  | |  |  | |  | *Neonectria major* | | **1** |  | |  |  |
|  | |  |  |  |  |  | *Leohumicola minima* | | **1** |  | |  | |  |  |  | |  |  | |  |  | |  | *Neonectria punicea* | | **4** |  | |  |  |
|  | |  |  |  |  |  | *Leohumicola sp. DAOM 230084* | | **1** |  | |  | |  |  |  | |  |  | |  |  | |  | *Ophiocordyceps crassispora* | | **41** |  | |  |  |
|  | |  |  |  |  |  | *Leohumicola verrucosa* | | **2** |  | |  | |  |  |  | |  |  | |  |  | |  | *Ophiocordyceps sinensis* | | **6** |  | |  |  |
|  | |  |  |  |  |  | *Leptodontidium sp. EF-60* | | **2** |  | |  | |  |  |  | |  |  | |  |  | |  | *Paracamarosporium hawaiiense* | | **1** |  | |  |  |
|  | |  |  |  |  |  | *Lophiostoma cynaroidis* | | **1** |  | |  | |  |  |  | |  |  | |  |  | |  | *Petrakia sp. ef08-038* | | **3** |  | |  |  |
|  | |  |  |  |  |  | *Lophiostoma macrostomoides* | | **1** |  | |  | |  |  |  | |  |  | |  |  | |  | *Pezicula corylina* | | **1** |  | |  |  |
|  | |  |  |  |  |  | *Mariannaea elegans* | | **3** |  | |  | |  |  |  | |  |  | |  |  | |  | *Pezicula ericae* | | **2** |  | |  |  |
|  | |  |  |  |  |  | *Massarina albocarnis* | | **1** |  | |  | |  |  |  | |  |  | |  |  | |  | *Pezicula frangulae* | | **1** |  | |  |  |
|  | |  |  |  |  |  | *Meliniomyces bicolor* | | **12** |  | |  | |  |  |  | |  |  | |  |  | |  | *Pezicula melanigena* | | **2** |  | |  |  |
|  | |  |  |  |  |  | *Meliniomyces variabilis* | | **7** |  | |  | |  |  |  | |  |  | |  |  | |  | *Pezoloma ericae* | | **1** |  | |  |  |
|  | |  |  |  |  |  | *Metapochonia suchlasporia* | | **1** |  | |  | |  |  |  | |  |  | |  |  | |  | *Phacidium grevilleae* | | **9** |  | |  |  |
|  | |  |  |  |  |  | *Metarhizium carneum* | | **5** |  | |  | |  |  |  | |  |  | |  |  | |  | *Phialea strobilina* | | **3** |  | |  |  |
|  | |  |  |  |  |  | *Microglossum viride* | | **1** |  | |  | |  |  |  | |  |  | |  |  | |  | *Phialocephala virens* | | **2** |  | |  |  |
|  | |  |  |  |  |  | *Microsphaeropsis arundinis* | | **1** |  | |  | |  |  |  | |  |  | |  |  | |  | *Phialophora sp. MLB-Phi* | | **1** |  | |  |  |
|  | |  |  |  |  |  | *Microsphaeropsis proteae* | | **4** |  | |  | |  |  |  | |  |  | |  |  | |  | *Phlyctema vagabunda* | | **1** |  | |  |  |
|  | |  |  |  |  |  | *Monilinia laxa* | | **5** |  | |  | |  |  |  | |  |  | |  |  | |  | *Phoma herbarum* | | **3** |  | |  |  |
|  | |  |  |  |  |  | *Monodictys arctica* | | **1** |  | |  | |  |  |  | |  |  | |  |  | |  | *Phoma sp. UM 186* | | **1** |  | |  |  |
|  | |  |  |  |  |  | *Mortierella sp. F121* | | **1** |  | |  | |  |  |  | |  |  | |  |  | |  | *Pilidium acerinum* | | **1** |  | |  |  |
|  | |  |  |  |  |  | *Mycochaetophora gentianae* | | **1** |  | |  | |  |  |  | |  |  | |  |  | |  | *Pilidium concavum* | | **5** |  | |  |  |
|  | |  |  |  |  |  | *Mycosymbioces mycenaphila* | | **15** |  | |  | |  |  |  | |  |  | |  |  | |  | *Podospora pleiospora* | | **1** |  | |  |  |
|  | |  |  |  |  |  | *Myxotrichum sp. 20081189* | | **1** |  | |  | |  |  |  | |  |  | |  |  | |  | *Preussia africana* | | **1** |  | |  |  |
|  | |  |  |  |  |  | *Nectria cinnabarina* | | **1** |  | |  | |  |  |  | |  |  | |  |  | |  | *Preussia minimoides* | | **2** |  | |  |  |
|  | |  |  |  |  |  | *Nectria sp.* | | **4** |  | |  | |  |  |  | |  |  | |  |  | |  | *Pseudaegerita sp. RJ-2015* | | **1** |  | |  |  |
|  | |  |  |  |  |  | *Neofabraea citricarpa* | | **1** |  | |  | |  |  |  | |  |  | |  |  | |  | *Sarimanas shirakamiense* | | **25** |  | |  |  |
|  | |  |  |  |  |  | *Neofabraea krawtzewii* | | **4** |  | |  | |  |  |  | |  |  | |  |  | |  | *Scleropezicula alnicola* | | **2** |  | |  |  |
|  | |  |  |  |  |  | *Neofabraea malicorticis* | | **1** |  | |  | |  |  |  | |  |  | |  |  | |  | *Scytalidium lignicola* | | **5** |  | |  |  |
|  | |  |  |  |  |  | *Neofabraea perennans* | | **2** |  | |  | |  |  |  | |  |  | |  |  | |  | *Serendipita vermifera* | | **1** |  | |  |  |
|  | |  |  |  |  |  | *Neonectria candida* | | **2** |  | |  | |  |  |  | |  |  | |  |  | |  | *Sphaeriothyrium filicinum* | | **1** |  | |  |  |
|  | |  |  |  |  |  | *Neonectria lugdunensis* | | **6** |  | |  | |  |  |  | |  |  | |  |  | |  | *Sphaerobolus iowensis* | | **3** |  | |  |  |
|  | |  |  |  |  |  | *Neonectria major* | | **1** |  | |  | |  |  |  | |  |  | |  |  | |  | *Spirosphaera floriformis* | | **25** |  | |  |  |
|  | |  |  |  |  |  | *Neonectria neomacrospora* | | **1** |  | |  | |  |  |  | |  |  | |  |  | |  | *Stagonosporopsis cucurbitacearum* | | **1** |  | |  |  |
|  | |  |  |  |  |  | *Neonectria punicea* | | **17** |  | |  | |  |  |  | |  |  | |  |  | |  | *Thelebolus sp. MUT 5300* | | **2** |  | |  |  |
|  | |  |  |  |  |  | *Ophiocordyceps crassispora* | | **111** |  | |  | |  |  |  | |  |  | |  |  | |  | *Thuemenidium atropurpureum* | | **6** |  | |  |  |
|  | |  |  |  |  |  | *Ophiocordyceps sinensis* | | **2** |  | |  | |  |  |  | |  |  | |  |  | |  | *Tolypocladium inegoense* | | **1** |  | |  |  |
|  | |  |  |  |  |  | *Ophiosphaerella herpotricha* | | **1** |  | |  | |  |  |  | |  |  | |  |  | |  | *Tolypocladium inflatum* | | **1** |  | |  |  |
|  | |  |  |  |  |  | *Paracamarosporium hawaiiense* | | **1** |  | |  | |  |  |  | |  |  | |  |  | |  | *Torrendiella andina* | | **2** |  | |  |  |
|  | |  |  |  |  |  | *Paraphaeosphaeria sporulosa* | | **1** |  | |  | |  |  |  | |  |  | |  |  | |  | *Truncatella angustata* | | **36** |  | |  |  |
|  | |  |  |  |  |  | *Parengyodontium album* | | **3** |  | |  | |  |  |  | |  |  | |  |  | |  | *Venturia hystrioides* | | **1** |  | |  |  |
|  | |  |  |  |  |  | *Periconia byssoides* | | **1** |  | |  | |  |  |  | |  |  | |  |  | |  | *Venturia lonicerae* | | **1** |  | |  |  |
|  | |  |  |  |  |  | *Pestalotiopsis maculiformans* | | **1** |  | |  | |  |  |  | |  |  | |  |  | |  | *Verrucoconiothyrium nitidae* | | **1** |  | |  |  |
|  | |  |  |  |  |  | *Petrakia aceris* | | **1** |  | |  | |  |  |  | |  |  | |  |  | |  | *Vestigium trifidum* | | **4** |  | |  |  |
|  | |  |  |  |  |  | *Petrakia sp. ef08-038* | | **1** |  | |  | |  |  |  | |  |  | |  |  | |  | *Vibrissea filisporia* | | **2** |  | |  |  |
|  | |  |  |  |  |  | *Pezicula aurantiaca* | | **1** |  | |  | |  |  |  | |  |  | |  |  | |  | *Vibrissea sp. PDD 99892* | | **1** |  | |  |  |
|  | |  |  |  |  |  | *Pezicula corylina* | | **1** |  | |  | |  |  |  | |  |  | |  |  | |  | *Vibrissea truncorum* | | **7** |  | |  |  |
|  | |  |  |  |  |  | *Pezicula ericae* | | **1** |  | |  | |  |  |  | |  |  | |  |  | |  | *Xenostigmina zilleri* | | **5** |  | |  |  |
|  | |  |  |  |  |  | *Pezicula eucrita* | | **1** |  | |  | |  |  |  | |  |  | |  |  | |  | *Yunnania penicillata* | | **1** |  | |  |  |
|  | |  |  |  |  |  | *Pezicula frangulae* | | **5** |  | |  | |  |  |  | |  |  | |  |  | |  |  | |  |  | |  |  |
|  | |  |  |  |  |  | *Pezicula melanigena* | | **4** |  | |  | |  |  |  | |  |  | |  |  | |  |  | |  |  | |  |  |
|  | |  |  |  |  |  | *Pezicula microspora* | | **3** |  | |  | |  |  |  | |  |  | |  |  | |  |  | |  |  | |  |  |
|  | |  |  |  |  |  | *Pezoloma ericae* | | **1** |  | |  | |  |  |  | |  |  | |  |  | |  |  | |  |  | |  |  |
|  | |  |  |  |  |  | *Phacidiopycnis washingtonensis* | | **5** |  | |  | |  |  |  | |  |  | |  |  | |  |  | |  |  | |  |  |
|  | |  |  |  |  |  | *Phacidium grevilleae* | | **51** |  | |  | |  |  |  | |  |  | |  |  | |  |  | |  |  | |  |  |
|  | |  |  |  |  |  | *Phialea strobilina* | | **6** |  | |  | |  |  |  | |  |  | |  |  | |  |  | |  |  | |  |  |
|  | |  |  |  |  |  | *Phialocephala fortinii* | | **1** |  | |  | |  |  |  | |  |  | |  |  | |  |  | |  |  | |  |  |
|  | |  |  |  |  |  | *Phialocephala virens* | | **6** |  | |  | |  |  |  | |  |  | |  |  | |  |  | |  |  | |  |  |
|  | |  |  |  |  |  | *Phialophora sp. MLB-Phi* | | **1** |  | |  | |  |  |  | |  |  | |  |  | |  |  | |  |  | |  |  |
|  | |  |  |  |  |  | *Phlyctema vagabunda* | | **18** |  | |  | |  |  |  | |  |  | |  |  | |  |  | |  |  | |  |  |
|  | |  |  |  |  |  | *Preussia minimoides* | | **2** |  | |  | |  |  |  | |  |  | |  |  | |  |  | |  |  | |  |  |
|  | |  |  |  |  |  | *Protoventuria alpina* | | **1** |  | |  | |  |  |  | |  |  | |  |  | |  |  | |  |  | |  |  |
|  | |  |  |  |  |  | *Pseudaegerita sp. RJ-2015* | | **1** |  | |  | |  |  |  | |  |  | |  |  | |  |  | |  |  | |  |  |
|  | |  |  |  |  |  | *Pseudogymnoascus pannorum* | | **1** |  | |  | |  |  |  | |  |  | |  |  | |  |  | |  |  | |  |  |
|  | |  |  |  |  |  | *Pseudogymnoascus sp. BL578* | | **1** |  | |  | |  |  |  | |  |  | |  |  | |  |  | |  |  | |  |  |
|  | |  |  |  |  |  | *Pseudogymnoascus sp. FI687* | | **3** |  | |  | |  |  |  | |  |  | |  |  | |  |  | |  |  | |  |  |
|  | |  |  |  |  |  | *Rhizodermea veluwensis* | | **1** |  | |  | |  |  |  | |  |  | |  |  | |  |  | |  |  | |  |  |
|  | |  |  |  |  |  | *Sarcostroma bisetulatum* | | **2** |  | |  | |  |  |  | |  |  | |  |  | |  |  | |  |  | |  |  |
|  | |  |  |  |  |  | *Sarimanas shirakamiense* | | **4** |  | |  | |  |  |  | |  |  | |  |  | |  |  | |  |  | |  |  |
|  | |  |  |  |  |  | *Scytalidium lignicola* | | **3** |  | |  | |  |  |  | |  |  | |  |  | |  |  | |  |  | |  |  |
|  | |  |  |  |  |  | *Spirosphaera floriformis* | | **50** |  | |  | |  |  |  | |  |  | |  |  | |  |  | |  |  | |  |  |
|  | |  |  |  |  |  | *Stagonosporopsis cucurbitacearum* | | **1** |  | |  | |  |  |  | |  |  | |  |  | |  |  | |  |  | |  |  |
|  | |  |  |  |  |  | *Thelebolus sp. MUT 5300* | | **3** |  | |  | |  |  |  | |  |  | |  |  | |  |  | |  |  | |  |  |
|  | |  |  |  |  |  | *Thuemenidium atropurpureum* | | **16** |  | |  | |  |  |  | |  |  | |  |  | |  |  | |  |  | |  |  |
|  | |  |  |  |  |  | *Thyronectria berolinensis* | | **1** |  | |  | |  |  |  | |  |  | |  |  | |  |  | |  |  | |  |  |
|  | |  |  |  |  |  | *Thyronectria lamyi* | | **1** |  | |  | |  |  |  | |  |  | |  |  | |  |  | |  |  | |  |  |
|  | |  |  |  |  |  | *Thyronectria sinopica* | | **1** |  | |  | |  |  |  | |  |  | |  |  | |  |  | |  |  | |  |  |
|  | |  |  |  |  |  | *Tilachlidium brachiatum* | | **1** |  | |  | |  |  |  | |  |  | |  |  | |  |  | |  |  | |  |  |
|  | |  |  |  |  |  | *Torrendiella andina* | | **6** |  | |  | |  |  |  | |  |  | |  |  | |  |  | |  |  | |  |  |
|  | |  |  |  |  |  | *Trichoderma paraviridescens* | | **1** |  | |  | |  |  |  | |  |  | |  |  | |  |  | |  |  | |  |  |
|  | |  |  |  |  |  | *Truncatella angustata* | | **259** |  | |  | |  |  |  | |  |  | |  |  | |  |  | |  |  | |  |  |
|  | |  |  |  |  |  | *Truncatella helichrysi* | | **3** |  | |  | |  |  |  | |  |  | |  |  | |  |  | |  |  | |  |  |
|  | |  |  |  |  |  | *Umbilicaria esculenta* | | **1** |  | |  | |  |  |  | |  |  | |  |  | |  |  | |  |  | |  |  |
|  | |  |  |  |  |  | *Verrucoconiothyrium nitidae* | | **2** |  | |  | |  |  |  | |  |  | |  |  | |  |  | |  |  | |  |  |
|  | |  |  |  |  |  | *Vestigium trifidum* | | **11** |  | |  | |  |  |  | |  |  | |  |  | |  |  | |  |  | |  |  |
|  | |  |  |  |  |  | *Vibrissea filisporia* | | **2** |  | |  | |  |  |  | |  |  | |  |  | |  |  | |  |  | |  |  |
|  | |  |  |  |  |  | *Vibrissea sp. PDD 99892* | | **2** |  | |  | |  |  |  | |  |  | |  |  | |  |  | |  |  | |  |  |
|  | |  |  |  |  |  | *Vibrissea truncorum* | | **5** |  | |  | |  |  |  | |  |  | |  |  | |  |  | |  |  | |  |  |
|  | |  |  |  |  |  | *Xenostigmina zilleri* | | **2** |  | |  | |  |  |  | |  |  | |  |  | |  |  | |  |  | |  |  |

(a)


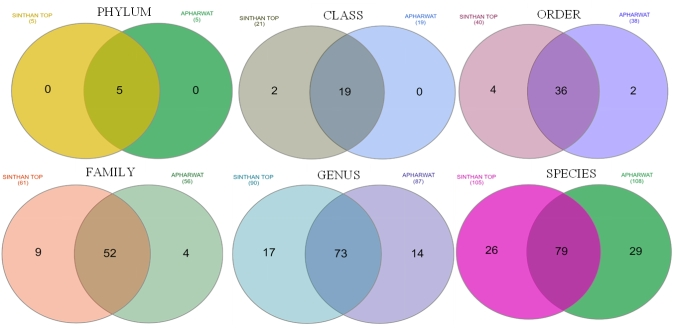


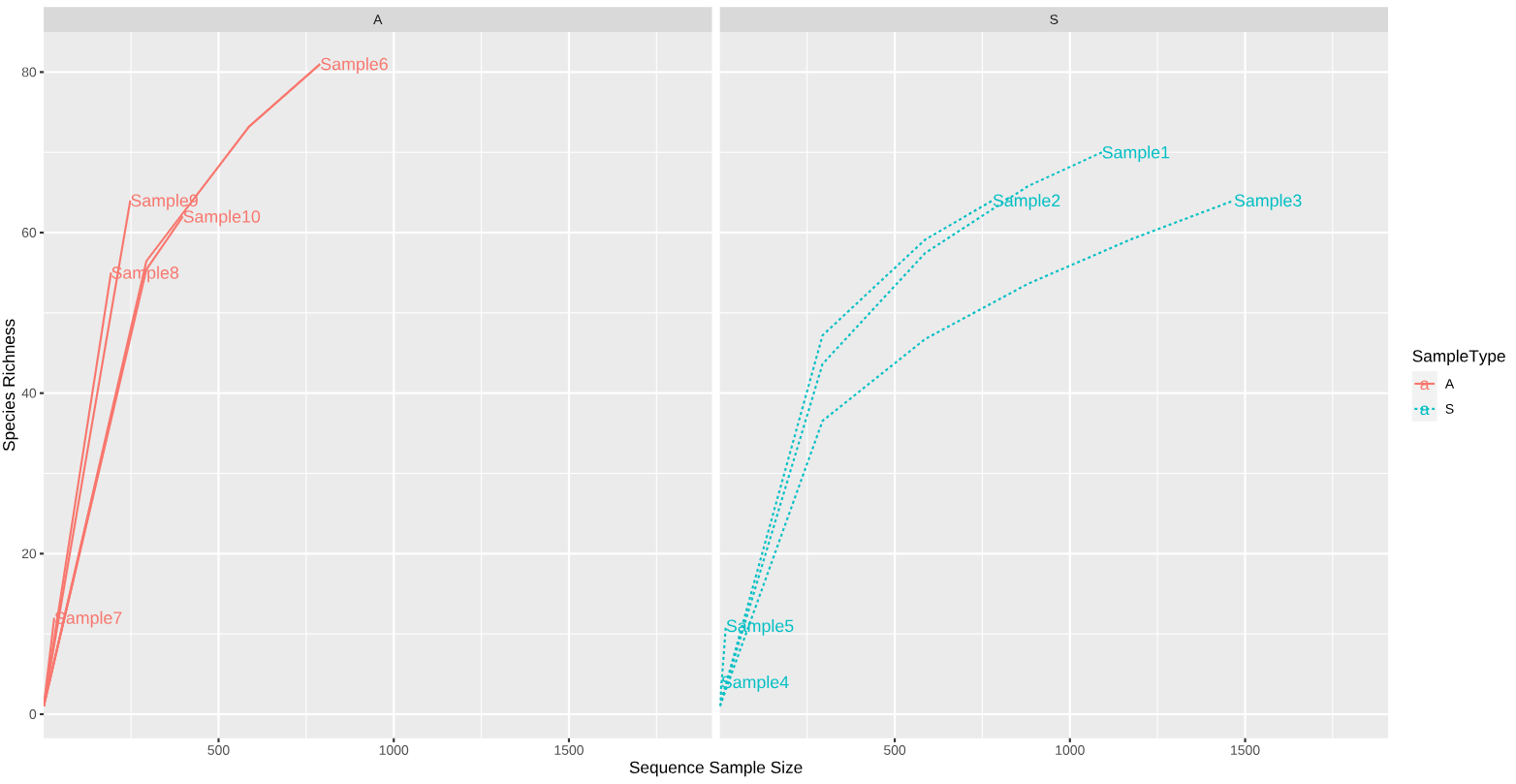
Supplementary Figure S2: Venn diagram shows the number of specific and shared taxa associated with *R. campanulatum*

Supplementary Figure S3: Rarefaction curves: sampling curves show the diversity of species within each sample across sites.

Supplementary Table S4: Diversity indices value for sample at each study site

| Study site | Sample site | Observed | Simpson | Shannon | Fisher | Chao1 |
| --- | --- | --- | --- | --- | --- | --- |
| **Sinthan Top** | Sample1 | 74 | 0.7248 | 2.42 | 17.91 | 90.5 |
|  | Sample 2 | 71 | 0.9212 | 3.15 | 18.93 | 92.08 |
|  | Sample 3 | 65 | 0.7803 | 2.415 | 13.92 | 111 |
|  | Sample 4 | 4 | 0.75 | 1.386 | 0 | 10 |
|  | Sample 5 | 13 | 0.81 | 2.164 | 16.1 | 79 |
| **Apharwat** | Sample 7 | 87 | 0.9489 | 3.513 | 24.87 | 112.8 |
|  | Sample 8 | 13 | 0.8359 | 2.127 | 8.155 | 31 |
|  | Sample 9 | 56 | 0.957 | 3.533 | 26.38 | 92.11 |
|  | Sample 10 | 66 | 0.9692 | 3.795 | 29.25 | 84.07 |
|  | Sample 11 | 64 | 0.953 | 3.466 | 21.49 | 83.46 |

Supplementary Table S5: FUNguild parsed classification

| S. No | Guild | Number of species |
| --- | --- | --- |
| 1 | Animal Pathogen | 9 |
| 2 | Ectomycorrhizal | 2 |
| 3 | Endophyte | 2 |
| 4 | Epiphyte | 1 |
| 5 | Lichenized | 2 |
| 6 | Unclassified fungi | 43 |
| 7 | Plant Pathogen | 15 |
| 8 | Undefined Saprotroph | 22 |
| 9 | Wood Saprotroph | 10 |
| 10 | Other fungi (more than one possible guild) | 28 |
